# Supplementary material for: Defining the biological basis of radiomic phenotypes in lung cancer
Source: eLife. 2017 Jul 21;6:e23421. doi: 10.7554/eLife.23421 (PMC5590809; doi:10.7554/eLife.23421)
Supplement: Figure 3—source data 1. — DOI: http://dx.doi.org/10.7554/eLife.23421.009 [file elife-23421-fig3-data1.pdf]

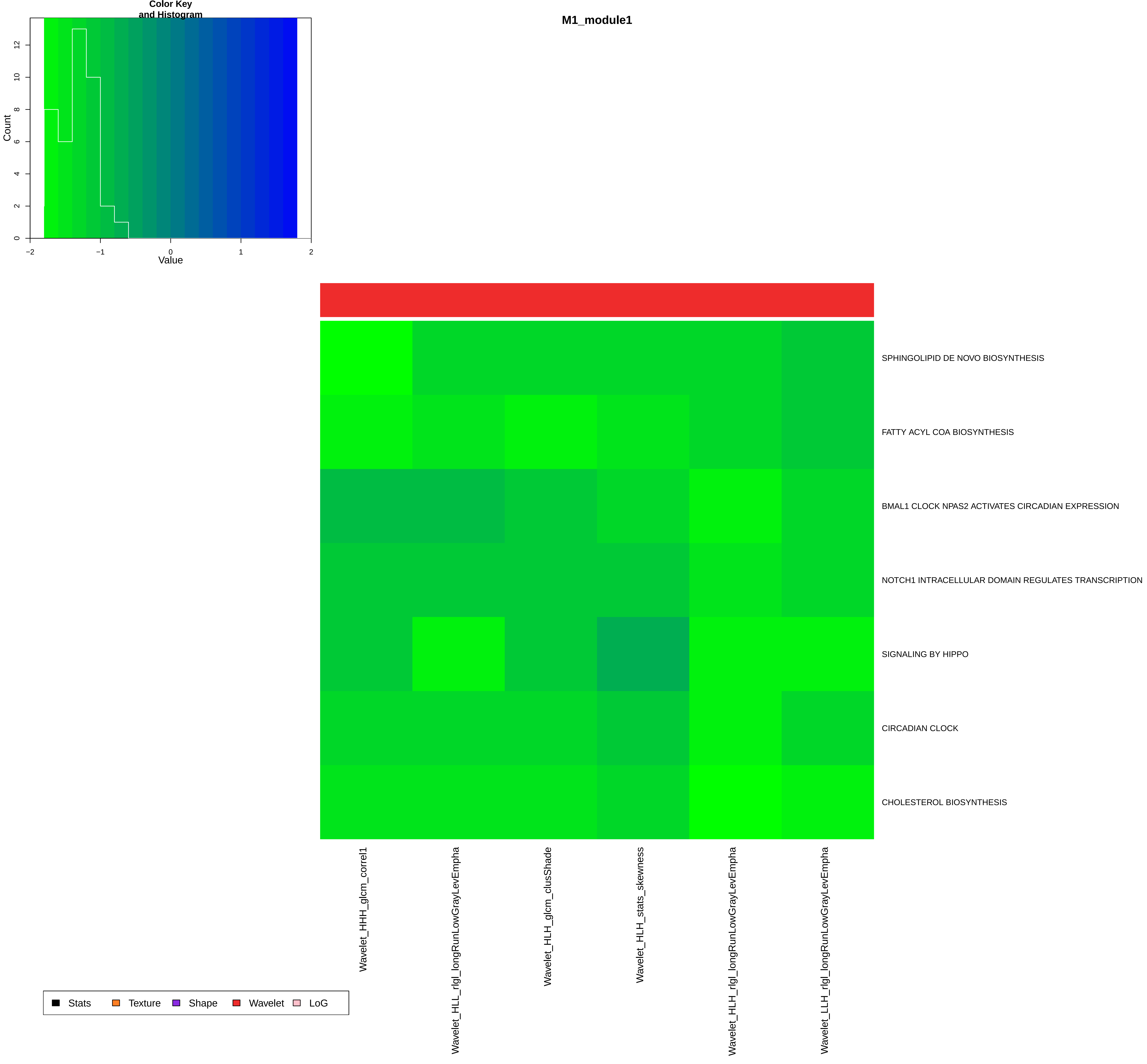
Color Key
and Histogram
Count
Value
M1_module1
SPHINGOLIPID DE NOVO BIOSYNTHESIS
FATTY ACYL COA BIOSYNTHESIS
BMAL1 CLOCK NPAS2 ACTIVATES CIRCADIAN EXPRESSION
NOTCH1 INTRACELLULAR DOMAIN REGULATES TRANSCRIPTION
SIGNALING BY HIPPO
CIRCADIAN CLOCK
CHOLESTEROL BIOSYNTHESIS
Wavelet_HHH_glcm_correl1
Wavelet_HLL_rlgl_longRunLowGrayLevEmpha
Wavelet_HLH_glcm_clusShade
Wavelet_HLH_stats_skewness
Wavelet_HLH_rlgl_longRunLowGrayLevEmpha
Wavelet_LLH_rlgl_longRunLowGrayLevEmpha
Stats
Texture
Shape
Wavelet
LoG

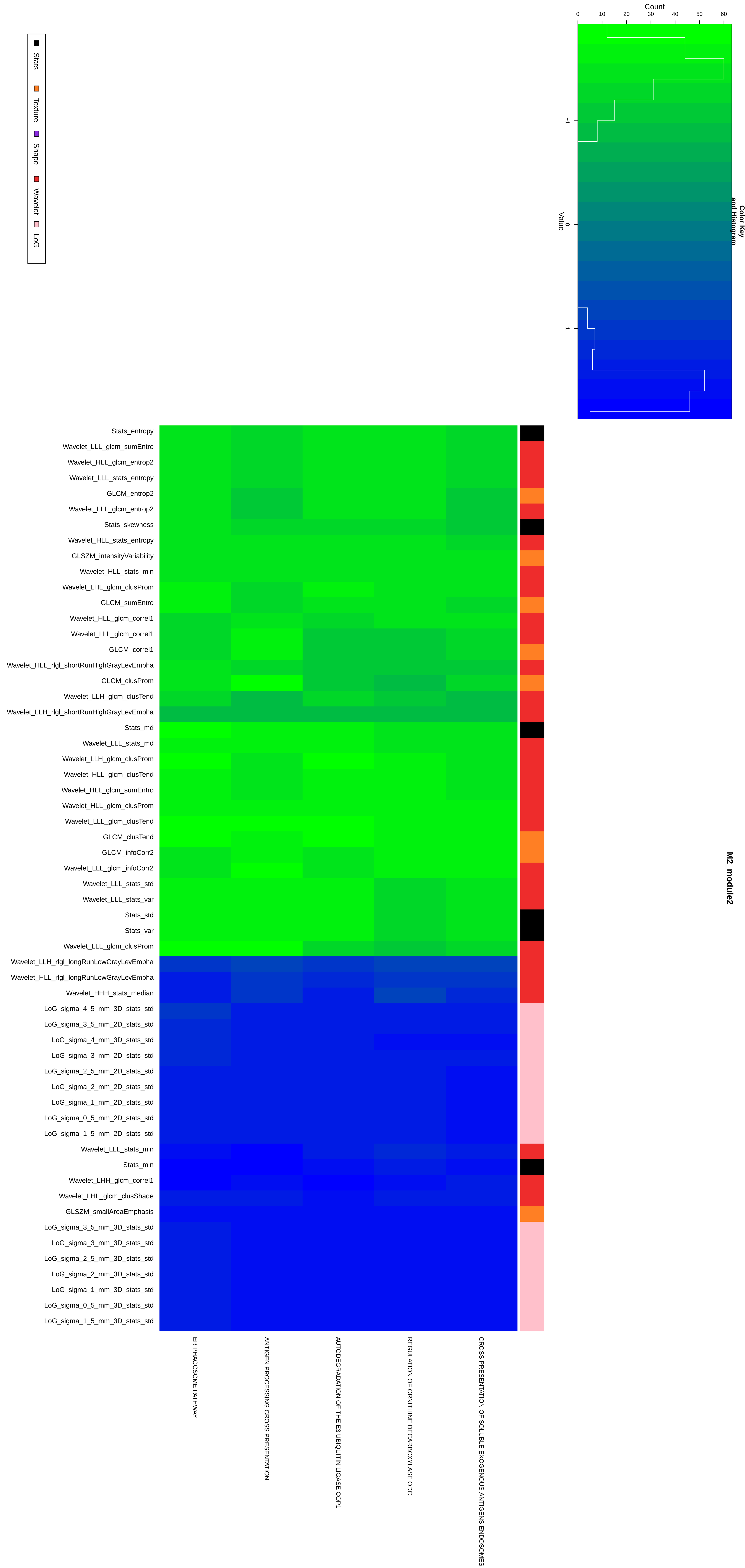
M2_module2
Color Key and Histogram
Count
0
10
20
30
40
50
60
Value
-1
0
1
Stats
Texture
Shape
Wavelet
LoG
Stats_entropy
Wavelet_LLL_glcm_sumEntro
Wavelet_HLL_glcm_entrop2
Wavelet_LLL_stats_entropy
GLCM_entrop2
Wavelet_LLL_glcm_entrop2
Stats_skewness
Wavelet_HLL_stats_entropy
GLSZM_intensityVariability
Wavelet_HLL_stats_min
Wavelet_LHL_glcm_clusProm
GLCM_sumEntro
Wavelet_HLL_glcm_correl1
Wavelet_LLL_glcm_correl1
GLCM_correl1
Wavelet_HLL_rlgl_shortRunHighGrayLevEmpha
GLCM_clusProm
Wavelet_LLH_glcm_clusTend
Wavelet_LLH_rlgl_shortRunHighGrayLevEmpha
Stats_md
Wavelet_LLL_stats_md
Wavelet_LLH_glcm_clusProm
Wavelet_HLL_glcm_clusTend
Wavelet_HLL_glcm_sumEntro
Wavelet_HLL_glcm_clusProm
Wavelet_LLL_glcm_clusTend
GLCM_clusTend
GLCM_infoCorr2
Wavelet_LLL_glcm_infoCorr2
Wavelet_LLL_stats_std
Wavelet_LLL_stats_var
Stats_std
Stats_var
Wavelet_LLL_glcm_clusProm
Wavelet_LLH_rlgl_longRunLowGrayLevEmpha
Wavelet_HLL_rlgl_longRunLowGrayLevEmpha
Wavelet_HHH_stats_median
LoG_sigma_4_5_mm_3D_stats_std
LoG_sigma_3_5_mm_2D_stats_std
LoG_sigma_4_mm_3D_stats_std
LoG_sigma_3_mm_2D_stats_std
LoG_sigma_2_5_mm_2D_stats_std
LoG_sigma_2_mm_2D_stats_std
LoG_sigma_1_mm_2D_stats_std
LoG_sigma_0_5_mm_2D_stats_std
LoG_sigma_1_5_mm_2D_stats_std
Wavelet_LLL_stats_min
Stats_min
Wavelet_LHH_glcm_correl1
Wavelet_LHL_glcm_clusShade
GLSZM_smallAreaEmphasis
LoG_sigma_3_5_mm_3D_stats_std
LoG_sigma_3_mm_3D_stats_std
LoG_sigma_2_5_mm_3D_stats_std
LoG_sigma_2_mm_3D_stats_std
LoG_sigma_1_mm_3D_stats_std
LoG_sigma_0_5_mm_3D_stats_std
LoG_sigma_1_5_mm_3D_stats_std
ER PHAGOSOME PATHWAY
ANTIGEN PROCESSING CROSS PRESENTATION
AUTODEGRADATION OF THE E3 UBIQUITIN LIGASE COP1
REGULATION OF ORNITHINE DECARBOXYLASE ODC
CROSS PRESENTATION OF SOLUBLE EXOGENOUS ANTIGENS ENDOSOMES

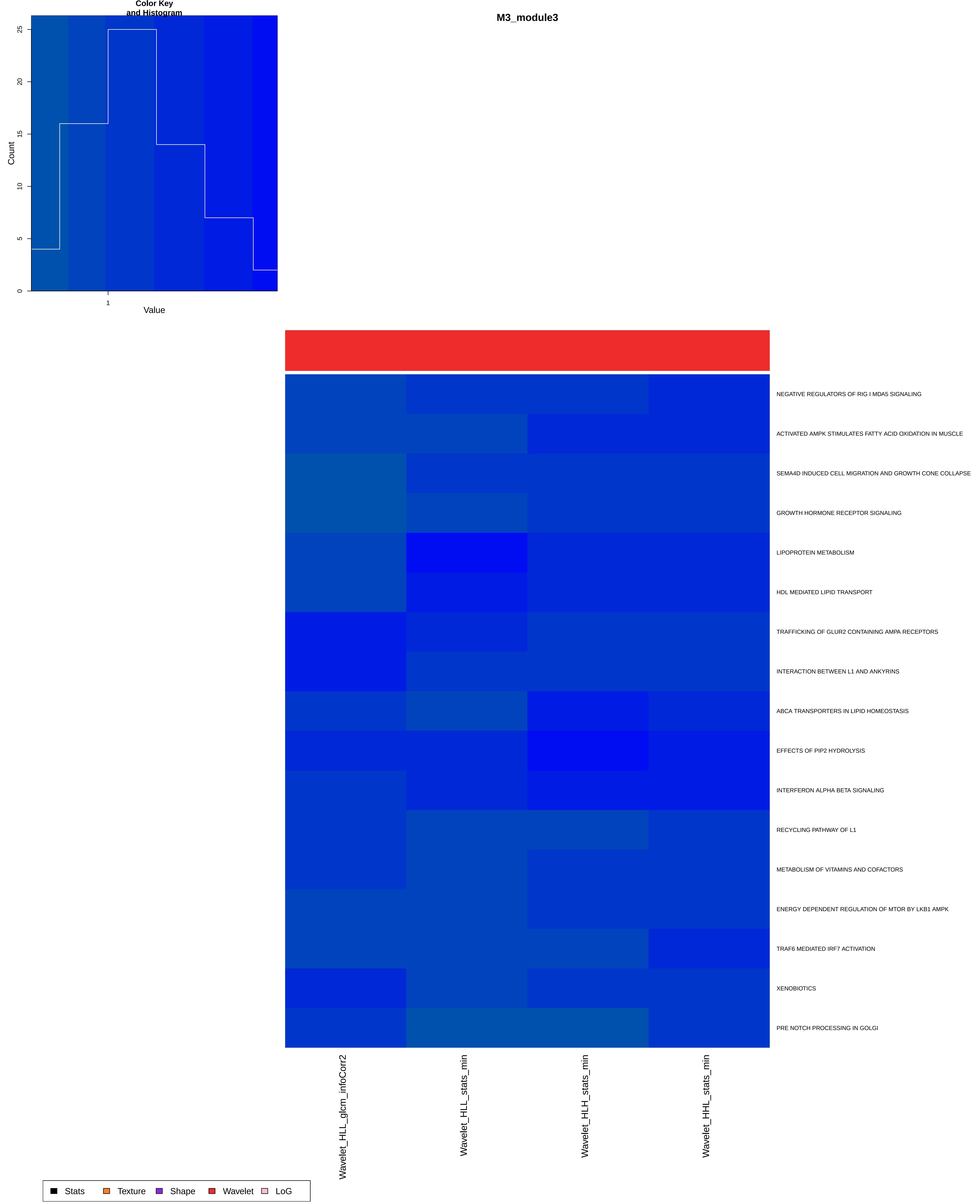
M3_module3
Color Key
and Histogram
Count
Value
0
5
10
15
20
25
1
NEGATIVE REGULATORS OF RIG I MDA5 SIGNALING
ACTIVATED AMPK STIMULATES FATTY ACID OXIDATION IN MUSCLE
SEMA4D INDUCED CELL MIGRATION AND GROWTH CONE COLLAPSE
GROWTH HORMONE RECEPTOR SIGNALING
LIPOPROTEIN METABOLISM
HDL MEDIATED LIPID TRANSPORT
TRAFFICKING OF GLUR2 CONTAINING AMPA RECEPTORS
INTERACTION BETWEEN L1 AND ANKYRINS
ABCA TRANSPORTERS IN LIPID HOMEOSTASIS
EFFECTS OF PIP2 HYDROLYSIS
INTERFERON ALPHA BETA SIGNALING
RECYCLING PATHWAY OF L1
METABOLISM OF VITAMINS AND COFACTORS
ENERGY DEPENDENT REGULATION OF MTOR BY LKB1 AMPK
TRAF6 MEDIATED IRF7 ACTIVATION
XENOBIOTICS
PRE NOTCH PROCESSING IN GOLGI
Wavelet_HLL_glcm_infoCorr2
Wavelet_HLL_stats_min
Wavelet_HLH_stats_min
Wavelet_HHL_stats_min
Stats
Texture
Shape
Wavelet
LoG

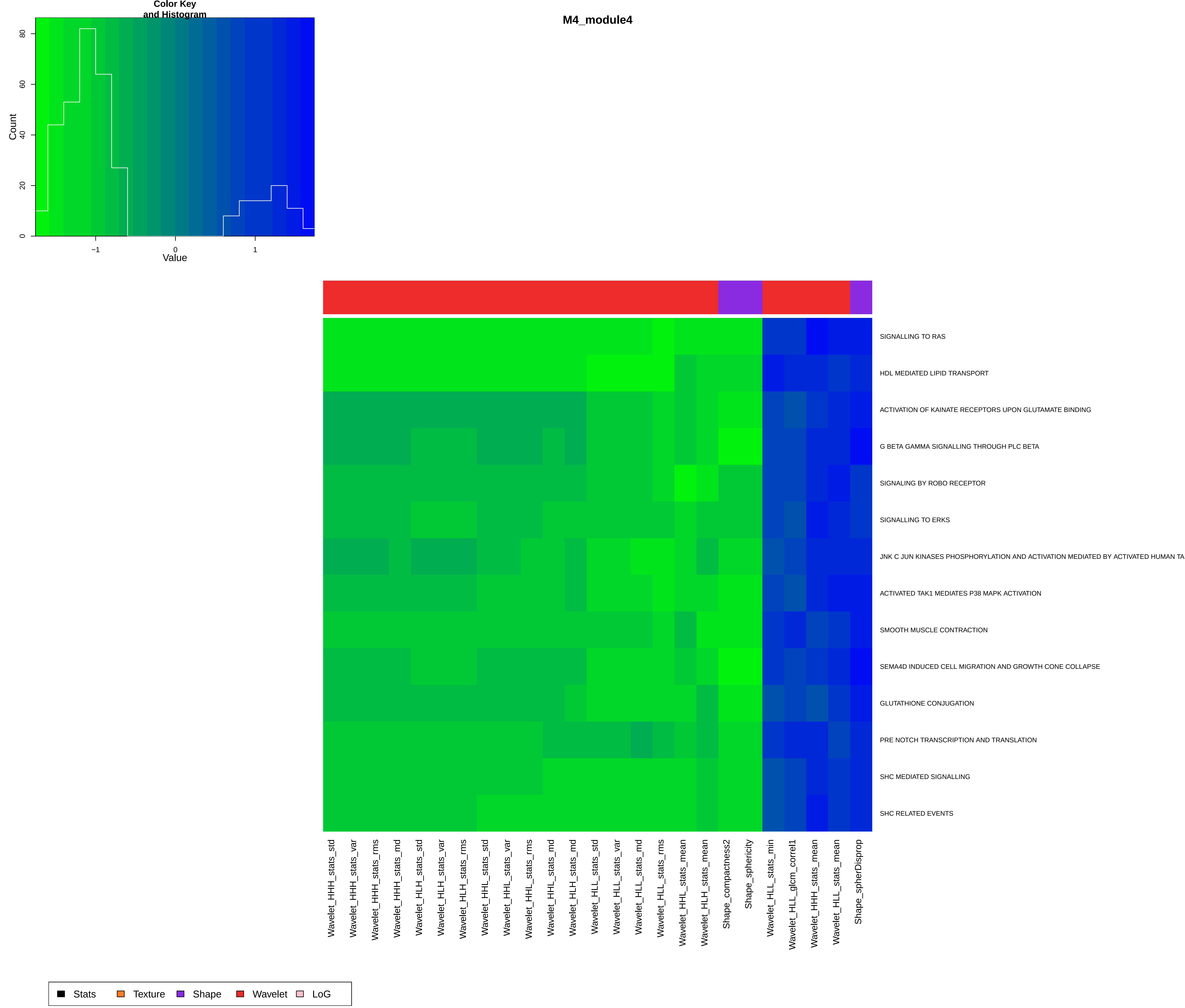
Color Key
and Histogram
Count
Value
M4_module4
SIGNALLING TO RAS
HDL MEDIATED LIPID TRANSPORT
ACTIVATION OF KAINATE RECEPTORS UPON GLUTAMATE BINDING
G BETA GAMMA SIGNALLING THROUGH PLC BETA
SIGNALING BY ROBO RECEPTOR
SIGNALLING TO ERKS
JNK C JUN KINASES PHOSPHORYLATION AND ACTIVATION MEDIATED BY ACTIVATED HUMAN TA
ACTIVATED TAK1 MEDIATES P38 MAPK ACTIVATION
SMOOTH MUSCLE CONTRACTION
SEMA4D INDUCED CELL MIGRATION AND GROWTH CONE COLLAPSE
GLUTATHIONE CONJUGATION
PRE NOTCH TRANSCRIPTION AND TRANSLATION
SHC MEDIATED SIGNALLING
SHC RELATED EVENTS
Wavelet_HHH_stats_std
Wavelet_HHH_stats_var
Wavelet_HHH_stats_rms
Wavelet_HHH_stats_md
Wavelet_HLH_stats_std
Wavelet_HLH_stats_var
Wavelet_HLH_stats_rms
Wavelet_HHL_stats_std
Wavelet_HHL_stats_var
Wavelet_HHL_stats_rms
Wavelet_HHL_stats_md
Wavelet_HLH_stats_md
Wavelet_HLL_stats_std
Wavelet_HLL_stats_var
Wavelet_HLL_stats_md
Wavelet_HLL_stats_rms
Wavelet_HHL_stats_mean
Wavelet_HLH_stats_mean
Shape_compactness2
Shape_sphericity
Wavelet_HLL_stats_min
Wavelet_HLL_glcm_correl1
Wavelet_HHH_stats_mean
Wavelet_HLL_stats_mean
Shape_spherDisprop
Stats
Texture
Shape
Wavelet
LoG

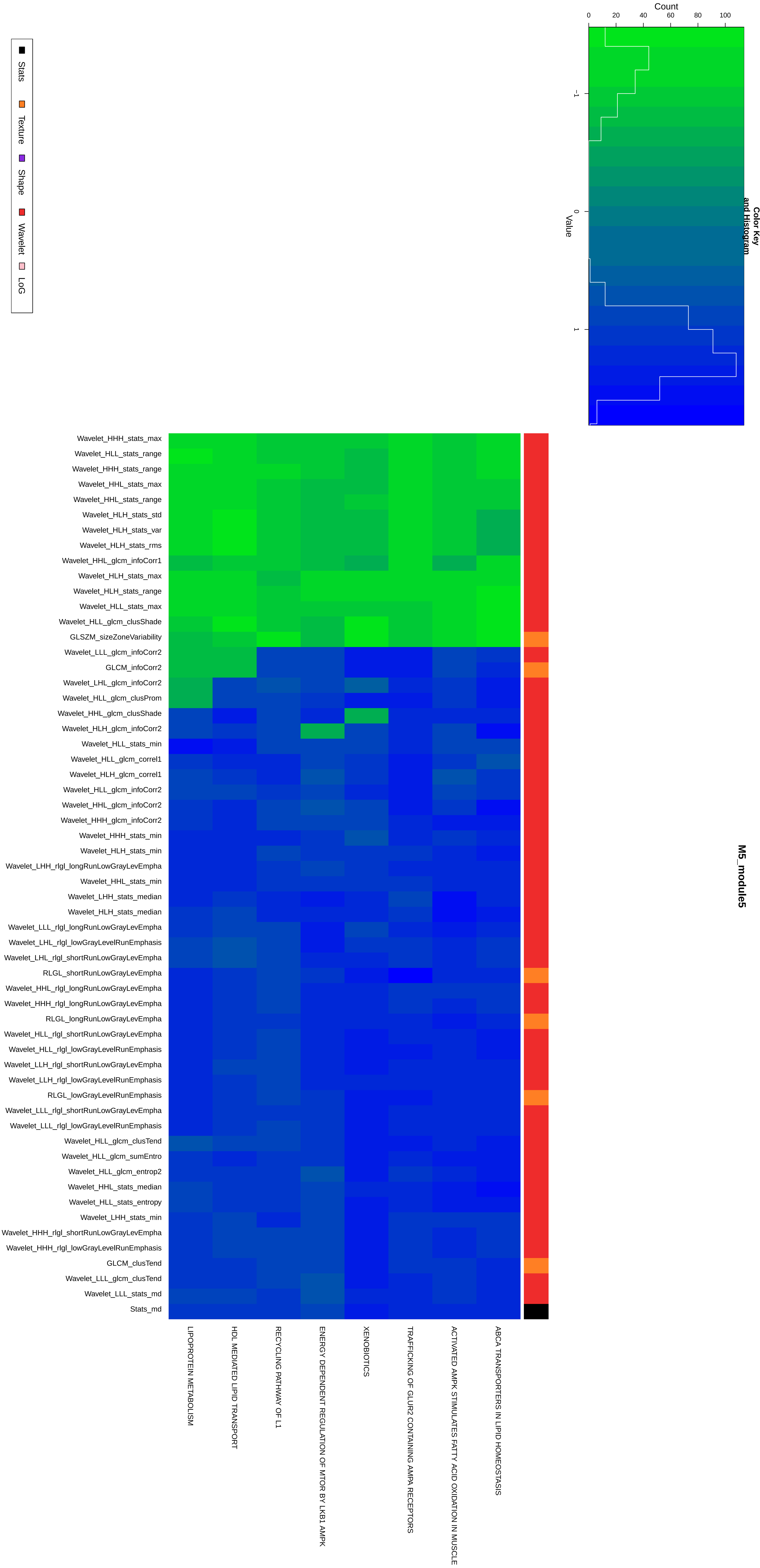
Color Key and Histogram
Count
0 20 40 60 80 100
Value
-1 0 1
Stats
Texture
Shape
Wavelet
LoG
M5_module5
Wavelet_HHH_stats_max
Wavelet_HLL_stats_range
Wavelet_HHH_stats_range
Wavelet_HHL_stats_max
Wavelet_HHL_stats_range
Wavelet_HLH_stats_std
Wavelet_HLH_stats_var
Wavelet_HLH_stats_rms
Wavelet_HHL_glcm_infoCorr1
Wavelet_HLH_stats_max
Wavelet_HLH_stats_range
Wavelet_HLL_stats_max
Wavelet_HLL_glcm_clusShade
GLSZM_sizeZoneVariability
Wavelet_LLL_glcm_infoCorr2
GLCM_infoCorr2
Wavelet_LHL_glcm_infoCorr2
Wavelet_HLL_glcm_clusProm
Wavelet_HHL_glcm_clusShade
Wavelet_HLH_glcm_infoCorr2
Wavelet_HLL_stats_min
Wavelet_HLL_glcm_correl1
Wavelet_HLH_glcm_correl1
Wavelet_HLL_glcm_infoCorr2
Wavelet_HHL_glcm_infoCorr2
Wavelet_HHH_glcm_infoCorr2
Wavelet_HHH_stats_min
Wavelet_HLH_stats_min
Wavelet_LHH_rlgl_longRunLowGrayLevEmpha
Wavelet_HHL_stats_min
Wavelet_LHH_stats_median
Wavelet_HLH_stats_median
Wavelet_LLL_rlgl_longRunLowGrayLevEmpha
Wavelet_LHL_rlgl_lowGrayLevelRunEmphasis
Wavelet_LHL_rlgl_shortRunLowGrayLevEmpha
RLGL_shortRunLowGrayLevEmpha
Wavelet_HHL_rlgl_longRunLowGrayLevEmpha
Wavelet_HHH_rlgl_longRunLowGrayLevEmpha
RLGL_longRunLowGrayLevEmpha
Wavelet_HLL_rlgl_shortRunLowGrayLevEmpha
Wavelet_HLL_rlgl_lowGrayLevelRunEmphasis
Wavelet_LLH_rlgl_shortRunLowGrayLevEmpha
Wavelet_LLH_rlgl_lowGrayLevelRunEmphasis
RLGL_lowGrayLevelRunEmphasis
Wavelet_LLL_rlgl_shortRunLowGrayLevEmpha
Wavelet_LLL_rlgl_lowGrayLevelRunEmphasis
Wavelet_HLL_glcm_clusTend
Wavelet_HLL_glcm_sumEntro
Wavelet_HLL_glcm_entrop2
Wavelet_HHL_stats_median
Wavelet_HLL_stats_entropy
Wavelet_LHH_stats_min
Wavelet_HHH_rlgl_shortRunLowGrayLevEmpha
Wavelet_HHH_rlgl_lowGrayLevelRunEmphasis
GLCM_clusTend
Wavelet_LLL_glcm_clusTend
Wavelet_LLL_stats_md
Stats_md
LIPOPROTEIN METABOLISM
HDL MEDIATED LIPID TRANSPORT
RECYCLING PATHWAY OF L1
ENERGY DEPENDENT REGULATION OF MTOR BY LKB1 AMPK
XENOBIOTICS
TRAFFICKING OF GLUR2 CONTAINING AMPA RECEPTORS
ACTIVATED AMPK STIMULATES FATTY ACID OXIDATION IN MUSCLE
ABCA TRANSPORTERS IN LIPID HOMEOSTASIS

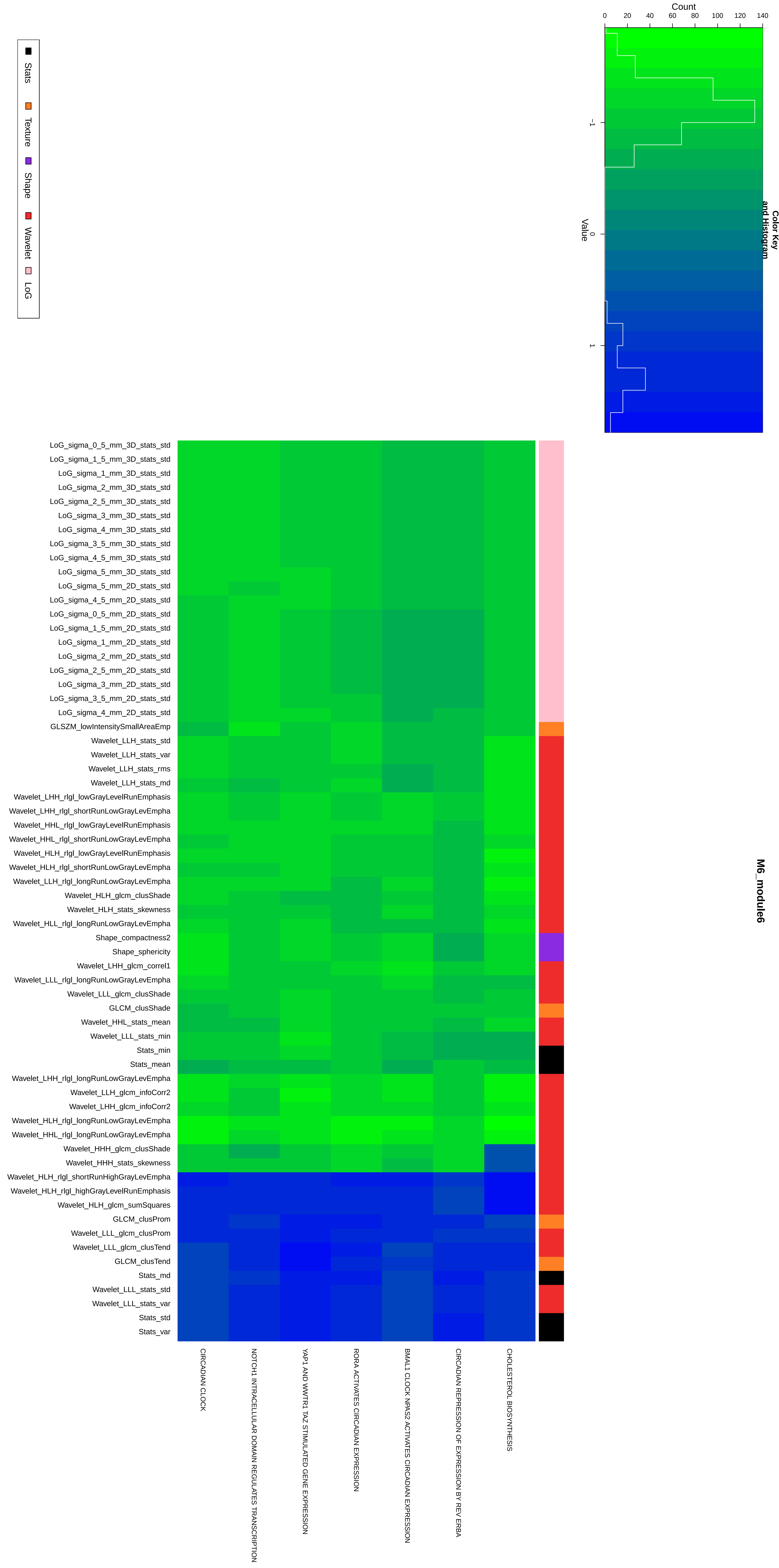
Stats
Texture
Shape
Wavelet
LoG
Color Key and Histogram
Count
0
20
40
60
80
100
120
140
Value
-1
0
1
M6_module6
LoG_sigma_0_5_mm_3D_stats_std
LoG_sigma_1_5_mm_3D_stats_std
LoG_sigma_1_mm_3D_stats_std
LoG_sigma_2_mm_3D_stats_std
LoG_sigma_2_5_mm_3D_stats_std
LoG_sigma_3_mm_3D_stats_std
LoG_sigma_4_mm_3D_stats_std
LoG_sigma_3_5_mm_3D_stats_std
LoG_sigma_4_5_mm_3D_stats_std
LoG_sigma_5_mm_3D_stats_std
LoG_sigma_5_mm_2D_stats_std
LoG_sigma_4_5_mm_2D_stats_std
LoG_sigma_0_5_mm_2D_stats_std
LoG_sigma_1_5_mm_2D_stats_std
LoG_sigma_1_mm_2D_stats_std
LoG_sigma_2_mm_2D_stats_std
LoG_sigma_2_5_mm_2D_stats_std
LoG_sigma_3_mm_2D_stats_std
LoG_sigma_3_5_mm_2D_stats_std
LoG_sigma_4_mm_2D_stats_std
GLSZM_lowIntensitySmallAreaEmp
Wavelet_LLH_stats_std
Wavelet_LLH_stats_var
Wavelet_LLH_stats_rms
Wavelet_LLH_stats_md
Wavelet_LHH_rlgl_lowGrayLevelRunEmphasis
Wavelet_LHH_rlgl_shortRunLowGrayLevEmpha
Wavelet_HHL_rlgl_lowGrayLevelRunEmphasis
Wavelet_HHL_rlgl_shortRunLowGrayLevEmpha
Wavelet_HLH_rlgl_lowGrayLevelRunEmphasis
Wavelet_HLH_rlgl_shortRunLowGrayLevEmpha
Wavelet_LLH_rlgl_longRunLowGrayLevEmpha
Wavelet_HLH_glcm_clusShade
Wavelet_HLH_stats_skewness
Wavelet_HLL_rlgl_longRunLowGrayLevEmpha
Shape_compactness2
Shape_sphericity
Wavelet_LHH_glcm_correl1
Wavelet_LLL_rlgl_longRunLowGrayLevEmpha
Wavelet_LLL_glcm_clusShade
GLCM_clusShade
Wavelet_HHL_stats_mean
Wavelet_LLL_stats_min
Stats_min
Stats_mean
Wavelet_LHH_rlgl_longRunLowGrayLevEmpha
Wavelet_LLH_glcm_infoCorr2
Wavelet_LHH_glcm_infoCorr2
Wavelet_HLH_rlgl_longRunLowGrayLevEmpha
Wavelet_HHL_rlgl_longRunLowGrayLevEmpha
Wavelet_HHH_glcm_clusShade
Wavelet_HHH_stats_skewness
Wavelet_HLH_rlgl_shortRunHighGrayLevEmpha
Wavelet_HLH_rlgl_highGrayLevelRunEmphasis
Wavelet_HLH_glcm_sumSquares
GLCM_clusProm
Wavelet_LLL_glcm_clusProm
Wavelet_LLL_glcm_clusTend
GLCM_clusTend
Stats_md
Wavelet_LLL_stats_std
Wavelet_LLL_stats_var
Stats_std
Stats_var
CIRCADIAN CLOCK
NOTCH1 INTRACELLULAR DOMAIN REGULATES TRANSCRIPTION
YAP1 AND WWTR1 TAZ STIMULATED GENE EXPRESSION
RORA ACTIVATES CIRCADIAN EXPRESSION
BMAL1 CLOCK NPAS2 ACTIVATES CIRCADIAN EXPRESSION
CIRCADIAN REPRESSION OF EXPRESSION BY REV ERBA
CHOLESTEROL BIOSYNTHESIS

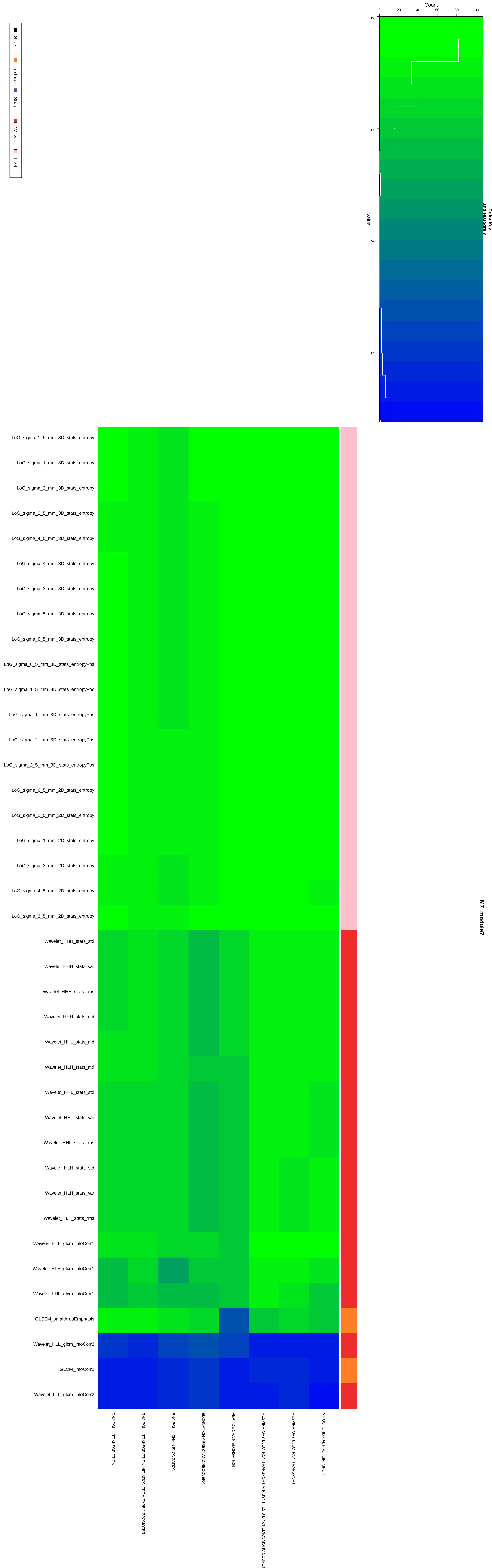
Color Key and Histogram
Count
0
20
40
60
80
100
-2
-1
0
1
Value
Stats
Texture
Shape
Wavelet
LoG
M7_module7
LoG_sigma_1_5_mm_3D_stats_entropy
LoG_sigma_1_mm_3D_stats_entropy
LoG_sigma_2_mm_3D_stats_entropy
LoG_sigma_2_5_mm_3D_stats_entropy
LoG_sigma_4_5_mm_3D_stats_entropy
LoG_sigma_4_mm_3D_stats_entropy
LoG_sigma_3_mm_3D_stats_entropy
LoG_sigma_5_mm_3D_stats_entropy
LoG_sigma_0_5_mm_3D_stats_entropy
LoG_sigma_0_5_mm_3D_stats_entropyPos
LoG_sigma_1_5_mm_3D_stats_entropyPos
LoG_sigma_1_mm_3D_stats_entropyPos
LoG_sigma_2_mm_3D_stats_entropyPos
LoG_sigma_2_5_mm_3D_stats_entropyPos
LoG_sigma_0_5_mm_2D_stats_entropy
LoG_sigma_1_5_mm_2D_stats_entropy
LoG_sigma_1_mm_2D_stats_entropy
LoG_sigma_3_mm_2D_stats_entropy
LoG_sigma_4_5_mm_2D_stats_entropy
LoG_sigma_3_5_mm_2D_stats_entropy
Wavelet_HHH_stats_std
Wavelet_HHH_stats_var
Wavelet_HHH_stats_rms
Wavelet_HHH_stats_md
Wavelet_HHL_stats_md
Wavelet_HLH_stats_md
Wavelet_HHL_stats_std
Wavelet_HHL_stats_var
Wavelet_HHL_stats_rms
Wavelet_HLH_stats_std
Wavelet_HLH_stats_var
Wavelet_HLH_stats_rms
Wavelet_HLL_glcm_infoCorr1
Wavelet_HLH_glcm_infoCorr1
Wavelet_LHL_glcm_infoCorr1
GLSZM_smallAreaEmphasis
Wavelet_HLL_glcm_infoCorr2
GLCM_infoCorr2
Wavelet_LLL_glcm_infoCorr2
RNA POL III TRANSCRIPTION
RNA POL III TRANSCRIPTION INITIATION FROM TYPE 2 PROMOTER
RNA POL III CHAIN ELONGATION
ELONGATION ARREST AND RECOVERY
PEPTIDE CHAIN ELONGATION
RESPIRATORY ELECTRON TRANSPORT ATP SYNTHESIS BY CHEMIOSMOTIC COUPLI
RESPIRATORY ELECTRON TRANSPORT
MITOCHONDRIAL PROTEIN IMPORT

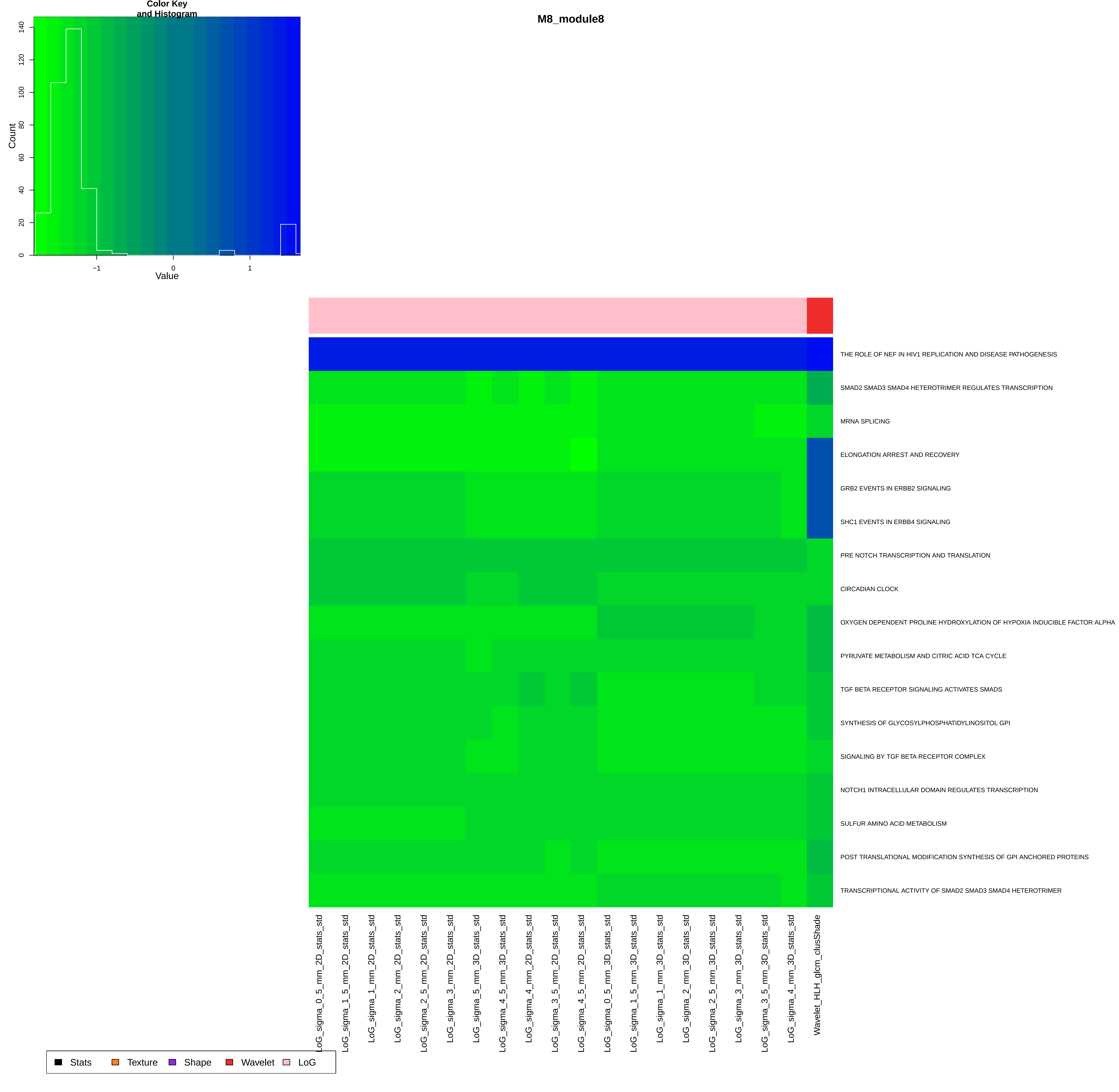

M8_module8
Color Key and Histogram
Count
Value
THE ROLE OF NEF IN HIV1 REPLICATION AND DISEASE PATHOGENESIS
SMAD2 SMAD3 SMAD4 HETEROTRIMER REGULATES TRANSCRIPTION
MRNA SPLICING
ELONGATION ARREST AND RECOVERY
GRB2 EVENTS IN ERBB2 SIGNALING
SHC1 EVENTS IN ERBB4 SIGNALING
PRE NOTCH TRANSCRIPTION AND TRANSLATION
CIRCADIAN CLOCK
OXYGEN DEPENDENT PROLINE HYDROXYLATION OF HYPOXIA INDUCIBLE FACTOR ALPHA
PYRUVATE METABOLISM AND CITRIC ACID TCA CYCLE
TGF BETA RECEPTOR SIGNALING ACTIVATES SMADS
SYNTHESIS OF GLYCOSYLPHOSPHATIDYLINOSITOL GPI
SIGNALING BY TGF BETA RECEPTOR COMPLEX
NOTCH1 INTRACELLULAR DOMAIN REGULATES TRANSCRIPTION
SULFUR AMINO ACID METABOLISM
POST TRANSLATIONAL MODIFICATION SYNTHESIS OF GPI ANCHORED PROTEINS
TRANSCRIPTIONAL ACTIVITY OF SMAD2 SMAD3 SMAD4 HETEROTRIMER
LoG_sigma_0_5_mm_2D_stats_std
LoG_sigma_1_5_mm_2D_stats_std
LoG_sigma_1_mm_2D_stats_std
LoG_sigma_2_mm_2D_stats_std
LoG_sigma_2_5_mm_2D_stats_std
LoG_sigma_3_mm_2D_stats_std
LoG_sigma_5_mm_3D_stats_std
LoG_sigma_4_5_mm_3D_stats_std
LoG_sigma_4_mm_2D_stats_std
LoG_sigma_3_5_mm_2D_stats_std
LoG_sigma_4_5_mm_2D_stats_std
LoG_sigma_0_5_mm_3D_stats_std
LoG_sigma_1_5_mm_3D_stats_std
LoG_sigma_1_mm_3D_stats_std
LoG_sigma_2_mm_3D_stats_std
LoG_sigma_2_5_mm_3D_stats_std
LoG_sigma_3_mm_3D_stats_std
LoG_sigma_3_5_mm_3D_stats_std
LoG_sigma_4_mm_3D_stats_std
Wavelet_HLH_glcm_clusShade
Stats
Texture
Shape
Wavelet
LoG

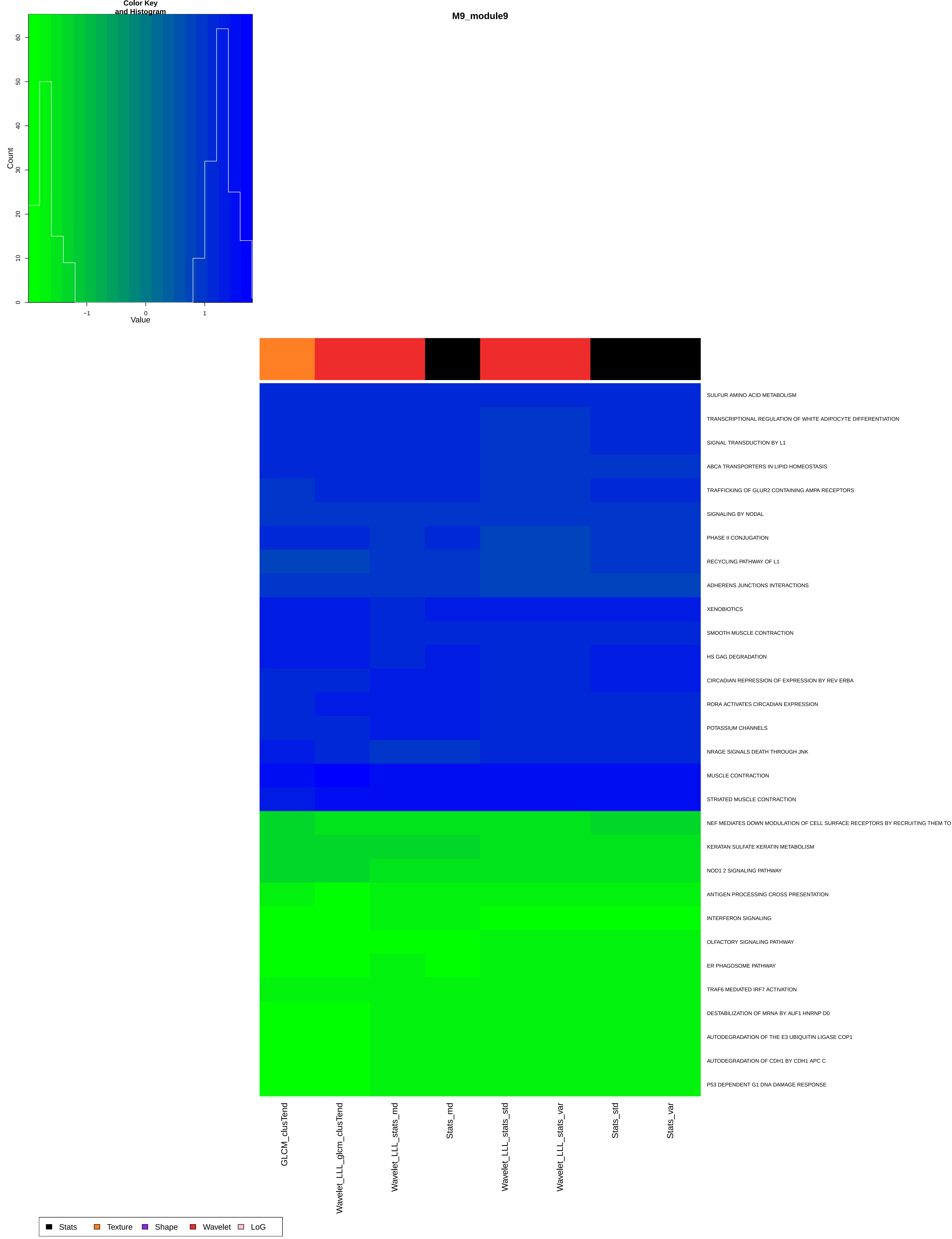

M9_module9
Color Key
and Histogram
Count
Value
0
10
20
30
40
50
60
−1
0
1
SULFUR AMINO ACID METABOLISM
TRANSCRIPTIONAL REGULATION OF WHITE ADIPOCYTE DIFFERENTIATION
SIGNAL TRANSDUCTION BY L1
ABCA TRANSPORTERS IN LIPID HOMEOSTASIS
TRAFFICKING OF GLUR2 CONTAINING AMPA RECEPTORS
SIGNALING BY NODAL
PHASE II CONJUGATION
RECYCLING PATHWAY OF L1
ADHERENS JUNCTIONS INTERACTIONS
XENOBIOTICS
SMOOTH MUSCLE CONTRACTION
HS GAG DEGRADATION
CIRCADIAN REPRESSION OF EXPRESSION BY REV ERBA
RORA ACTIVATES CIRCADIAN EXPRESSION
POTASSIUM CHANNELS
NRAGE SIGNALS DEATH THROUGH JNK
MUSCLE CONTRACTION
STRIATED MUSCLE CONTRACTION
NEF MEDIATES DOWN MODULATION OF CELL SURFACE RECEPTORS BY RECRUITING THEM TO
KERATAN SULFATE KERATIN METABOLISM
NOD1 2 SIGNALING PATHWAY
ANTIGEN PROCESSING CROSS PRESENTATION
INTERFERON SIGNALING
OLFACTORY SIGNALING PATHWAY
ER PHAGOSOME PATHWAY
TRAF6 MEDIATED IRF7 ACTIVATION
DESTABILIZATION OF MRNA BY AUF1 HNRNP D0
AUTODEGRADATION OF THE E3 UBIQUITIN LIGASE COP1
AUTODEGRADATION OF CDH1 BY CDH1 APC C
P53 DEPENDENT G1 DNA DAMAGE RESPONSE
GLCM_clusTend
Wavelet_LLL_glcm_clusTend
Wavelet_LLL_stats_md
Stats_md
Wavelet_LLL_stats_std
Wavelet_LLL_stats_var
Stats_std
Stats_var
Stats
Texture
Shape
Wavelet
LoG

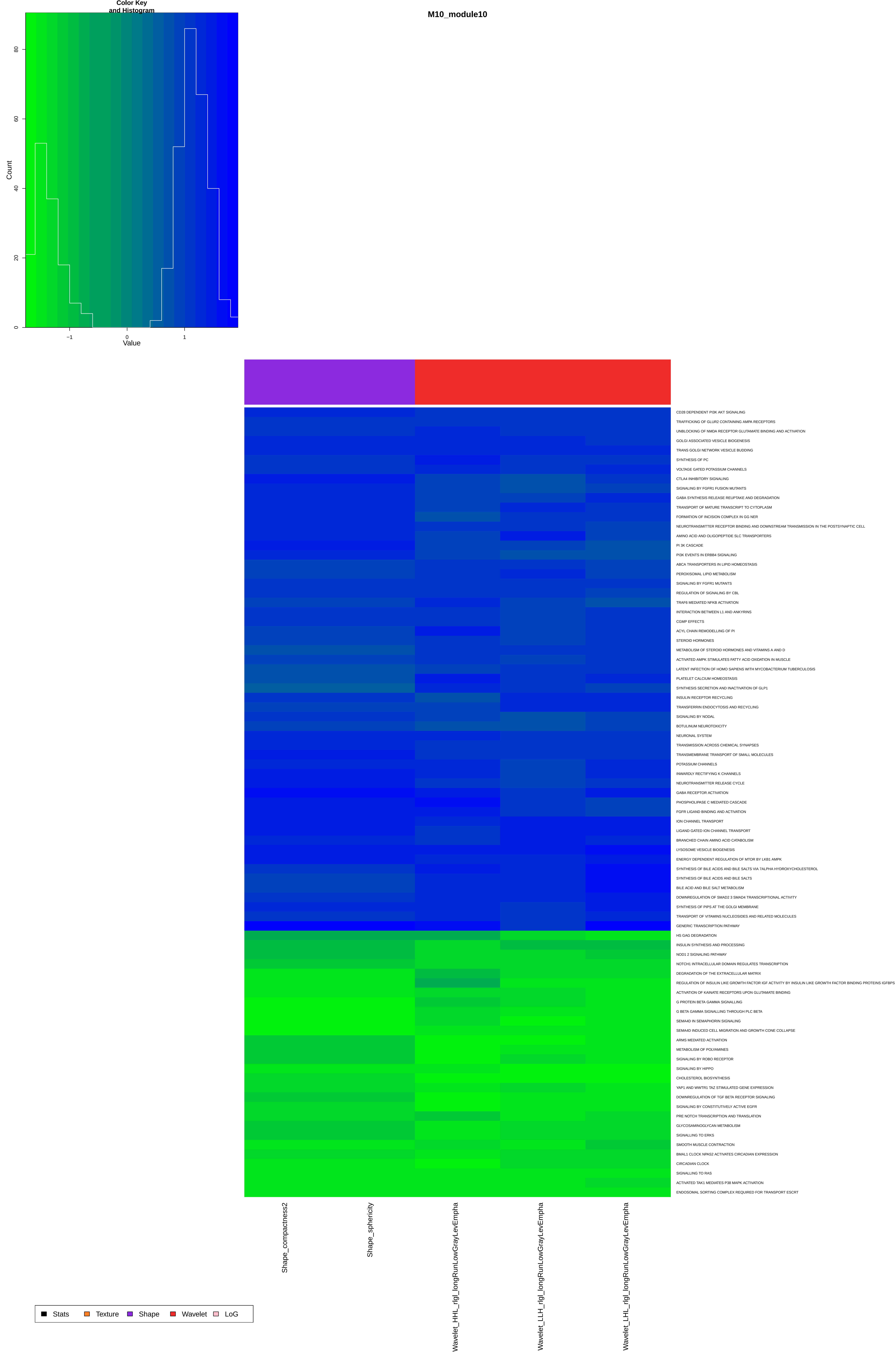

Color Key
and Histogram
Count
Value
M10_module10
CD28 DEPENDENT PI3K AKT SIGNALING
TRAFFICKING OF GLUR2 CONTAINING AMPA RECEPTORS
UNBLOCKING OF NMDA RECEPTOR GLUTAMATE BINDING AND ACTIVATION
GOLGI ASSOCIATED VESICLE BIOGENESIS
TRANS GOLGI NETWORK VESICLE BUDDING
SYNTHESIS OF PC
VOLTAGE GATED POTASSIUM CHANNELS
CTLA4 INHIBITORY SIGNALING
SIGNALING BY FGFR1 FUSION MUTANTS
GABA SYNTHESIS RELEASE REUPTAKE AND DEGRADATION
TRANSPORT OF MATURE TRANSCRIPT TO CYTOPLASM
FORMATION OF INCISION COMPLEX IN GG NER
NEUROTRANSMITTER RECEPTOR BINDING AND DOWNSTREAM TRANSMISSION IN THE POSTSYNAPTIC CELL
AMINO ACID AND OLIGOPEPTIDE SLC TRANSPORTERS
PI 3K CASCADE
PI3K EVENTS IN ERBB4 SIGNALING
ABCA TRANSPORTERS IN LIPID HOMEOSTASIS
PEROXISOMAL LIPID METABOLISM
SIGNALING BY FGFR1 MUTANTS
REGULATION OF SIGNALING BY CBL
TRAF6 MEDIATED NFKB ACTIVATION
INTERACTION BETWEEN L1 AND ANKYRINS
CGMP EFFECTS
ACYL CHAIN REMODELLING OF PI
STEROID HORMONES
METABOLISM OF STEROID HORMONES AND VITAMINS A AND D
ACTIVATED AMPK STIMULATES FATTY ACID OXIDATION IN MUSCLE
LATENT INFECTION OF HOMO SAPIENS WITH MYCOBACTERIUM TUBERCULOSIS
PLATELET CALCIUM HOMEOSTASIS
SYNTHESIS SECRETION AND INACTIVATION OF GLP1
INSULIN RECEPTOR RECYCLING
TRANSFERRIN ENDOCYTOSIS AND RECYCLING
SIGNALING BY NODAL
BOTULINUM NEUROTOXICITY
NEURONAL SYSTEM
TRANSMISSION ACROSS CHEMICAL SYNAPSES
TRANSMEMBRANE TRANSPORT OF SMALL MOLECULES
POTASSIUM CHANNELS
INWARDLY RECTIFYING K CHANNELS
NEUROTRANSMITTER RELEASE CYCLE
GABA RECEPTOR ACTIVATION
PHOSPHOLIPASE C MEDIATED CASCADE
FGFR LIGAND BINDING AND ACTIVATION
ION CHANNEL TRANSPORT
LIGAND GATED ION CHANNEL TRANSPORT
BRANCHED CHAIN AMINO ACID CATABOLISM
LYSOSOME VESICLE BIOGENESIS
ENERGY DEPENDENT REGULATION OF MTOR BY LKB1 AMPK
SYNTHESIS OF BILE ACIDS AND BILE SALTS VIA 7ALPHA HYDROXYCHOLESTEROL
SYNTHESIS OF BILE ACIDS AND BILE SALTS
BILE ACID AND BILE SALT METABOLISM
DOWNREGULATION OF SMAD2 3 SMAD4 TRANSCRIPTIONAL ACTIVITY
SYNTHESIS OF PIPS AT THE GOLGI MEMBRANE
TRANSPORT OF VITAMINS NUCLEOSIDES AND RELATED MOLECULES
GENERIC TRANSCRIPTION PATHWAY
HS GAG DEGRADATION
INSULIN SYNTHESIS AND PROCESSING
NOD1 2 SIGNALING PATHWAY
NOTCH1 INTRACELLULAR DOMAIN REGULATES TRANSCRIPTION
DEGRADATION OF THE EXTRACELLULAR MATRIX
REGULATION OF INSULIN LIKE GROWTH FACTOR IGF ACTIVITY BY INSULIN LIKE GROWTH FACTOR BINDING PROTEINS IGFBPS
ACTIVATION OF KAINATE RECEPTORS UPON GLUTAMATE BINDING
G PROTEIN BETA GAMMA SIGNALLING
G BETA GAMMA SIGNALLING THROUGH PLC BETA
SEMA4D IN SEMAPHORIN SIGNALING
SEMA4D INDUCED CELL MIGRATION AND GROWTH CONE COLLAPSE
ARMS MEDIATED ACTIVATION
METABOLISM OF POLYAMINES
SIGNALING BY ROBO RECEPTOR
SIGNALING BY HIPPO
CHOLESTEROL BIOSYNTHESIS
YAP1 AND WWTR1 TAZ STIMULATED GENE EXPRESSION
DOWNREGULATION OF TGF BETA RECEPTOR SIGNALING
SIGNALING BY CONSTITUTIVELY ACTIVE EGFR
PRE NOTCH TRANSCRIPTION AND TRANSLATION
GLYCOSAMINOGLYCAN METABOLISM
SIGNALLING TO ERKS
SMOOTH MUSCLE CONTRACTION
BMAL1 CLOCK NPAS2 ACTIVATES CIRCADIAN EXPRESSION
CIRCADIAN CLOCK
SIGNALLING TO RAS
ACTIVATED TAK1 MEDIATES P38 MAPK ACTIVATION
ENDOSOMAL SORTING COMPLEX REQUIRED FOR TRANSPORT ESCRT
Shape_compactness2
Shape_sphericity
Wavelet_HHL_rlgl_longRunLowGrayLevEmpha
Wavelet_LLH_rlgl_longRunLowGrayLevEmpha
Wavelet_LHL_rlgl_longRunLowGrayLevEmpha
Stats
Texture
Shape
Wavelet
LoG

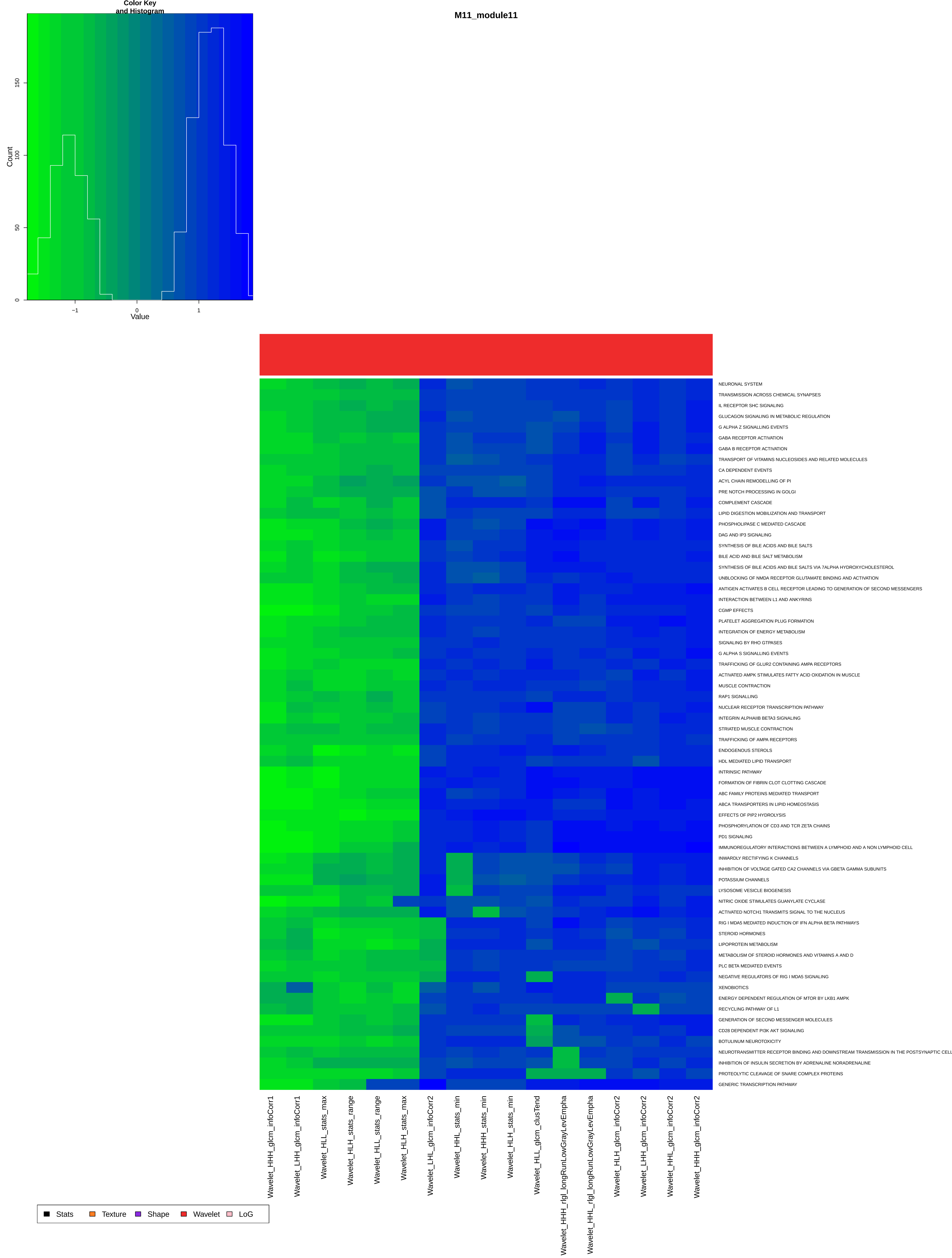
M11_module11
Color Key and Histogram
Count
Value
0
50
100
150
−1
0
1
Stats
Texture
Shape
Wavelet
LoG
NEURONAL SYSTEM
TRANSMISSION ACROSS CHEMICAL SYNAPSES
IL RECEPTOR SHC SIGNALING
GLUCAGON SIGNALING IN METABOLIC REGULATION
G ALPHA Z SIGNALLING EVENTS
GABA RECEPTOR ACTIVATION
GABA B RECEPTOR ACTIVATION
TRANSPORT OF VITAMINS NUCLEOSIDES AND RELATED MOLECULES
CA DEPENDENT EVENTS
ACYL CHAIN REMODELLING OF PI
PRE NOTCH PROCESSING IN GOLGI
COMPLEMENT CASCADE
LIPID DIGESTION MOBILIZATION AND TRANSPORT
PHOSPHOLIPASE C MEDIATED CASCADE
DAG AND IP3 SIGNALING
SYNTHESIS OF BILE ACIDS AND BILE SALTS
BILE ACID AND BILE SALT METABOLISM
SYNTHESIS OF BILE ACIDS AND BILE SALTS VIA 7ALPHA HYDROXYCHOLESTEROL
UNBLOCKING OF NMDA RECEPTOR GLUTAMATE BINDING AND ACTIVATION
ANTIGEN ACTIVATES B CELL RECEPTOR LEADING TO GENERATION OF SECOND MESSENGERS
INTERACTION BETWEEN L1 AND ANKYRINS
CGMP EFFECTS
PLATELET AGGREGATION PLUG FORMATION
INTEGRATION OF ENERGY METABOLISM
SIGNALING BY RHO GTPASES
G ALPHA S SIGNALLING EVENTS
TRAFFICKING OF GLUR2 CONTAINING AMPA RECEPTORS
ACTIVATED AMPK STIMULATES FATTY ACID OXIDATION IN MUSCLE
MUSCLE CONTRACTION
RAP1 SIGNALLING
NUCLEAR RECEPTOR TRANSCRIPTION PATHWAY
INTEGRIN ALPHAIIB BETA3 SIGNALING
STRIATED MUSCLE CONTRACTION
TRAFFICKING OF AMPA RECEPTORS
ENDOGENOUS STEROLS
HDL MEDIATED LIPID TRANSPORT
INTRINSIC PATHWAY
FORMATION OF FIBRIN CLOT CLOTTING CASCADE
ABC FAMILY PROTEINS MEDIATED TRANSPORT
ABCA TRANSPORTERS IN LIPID HOMEOSTASIS
EFFECTS OF PIP2 HYDROLYSIS
PHOSPHORYLATION OF CD3 AND TCR ZETA CHAINS
PD1 SIGNALING
IMMUNOREGULATORY INTERACTIONS BETWEEN A LYMPHOID AND A NON LYMPHOID CELL
INWARDLY RECTIFYING K CHANNELS
INHIBITION OF VOLTAGE GATED CA2 CHANNELS VIA GBETA GAMMA SUBUNITS
POTASSIUM CHANNELS
LYSOSOME VESICLE BIOGENESIS
NITRIC OXIDE STIMULATES GUANYLATE CYCLASE
ACTIVATED NOTCH1 TRANSMITS SIGNAL TO THE NUCLEUS
RIG I MDA5 MEDIATED INDUCTION OF IFN ALPHA BETA PATHWAYS
STEROID HORMONES
LIPOPROTEIN METABOLISM
METABOLISM OF STEROID HORMONES AND VITAMINS A AND D
PLC BETA MEDIATED EVENTS
NEGATIVE REGULATORS OF RIG I MDA5 SIGNALING
XENOBIOTICS
ENERGY DEPENDENT REGULATION OF MTOR BY LKB1 AMPK
RECYCLING PATHWAY OF L1
GENERATION OF SECOND MESSENGER MOLECULES
CD28 DEPENDENT PI3K AKT SIGNALING
BOTULINUM NEUROTOXICITY
NEUROTRANSMITTER RECEPTOR BINDING AND DOWNSTREAM TRANSMISSION IN THE POSTSYNAPTIC CELL
INHIBITION OF INSULIN SECRETION BY ADRENALINE NORADRENALINE
PROTEOLYTIC CLEAVAGE OF SNARE COMPLEX PROTEINS
GENERIC TRANSCRIPTION PATHWAY
Wavelet_HHH_glcm_infoCorr1
Wavelet_LHH_glcm_infoCorr1
Wavelet_HLL_stats_max
Wavelet_HLH_stats_range
Wavelet_HLL_stats_range
Wavelet_HLH_stats_max
Wavelet_LHL_glcm_infoCorr2
Wavelet_HHL_stats_min
Wavelet_HHH_stats_min
Wavelet_HLH_stats_min
Wavelet_HLL_glcm_clusTend
Wavelet_HHH_rlgl_longRunLowGrayLevEmpha
Wavelet_HHL_rlgl_longRunLowGrayLevEmpha
Wavelet_HLH_glcm_infoCorr2
Wavelet_LHH_glcm_infoCorr2
Wavelet_HHL_glcm_infoCorr2
Wavelet_HHH_glcm_infoCorr2

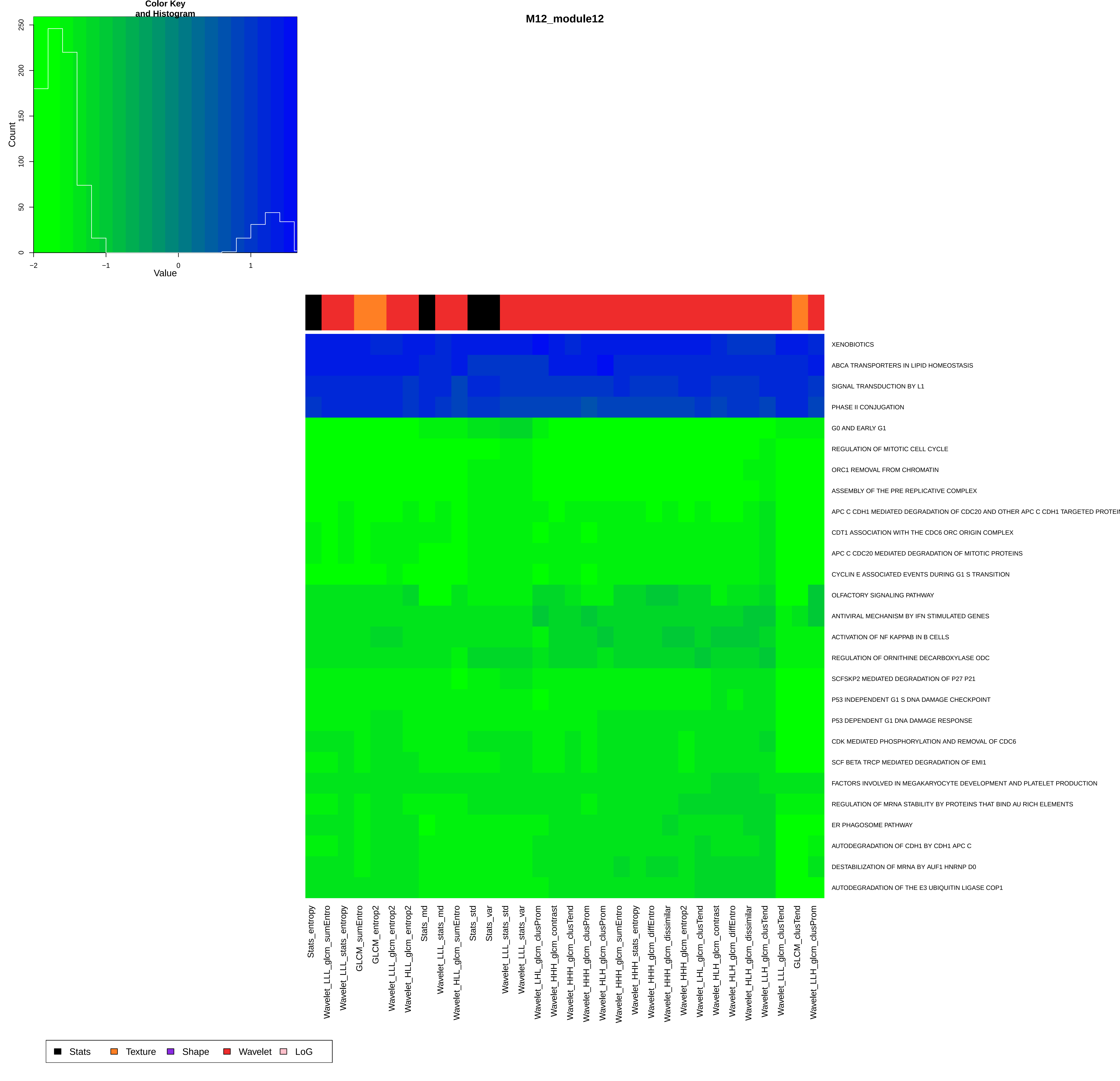
Color Key
and Histogram
Count
Value
0
50
100
150
200
250
−2
−1
0
1
M12_module12
XENOBIOTICS
ABCA TRANSPORTERS IN LIPID HOMEOSTASIS
SIGNAL TRANSDUCTION BY L1
PHASE II CONJUGATION
G0 AND EARLY G1
REGULATION OF MITOTIC CELL CYCLE
ORC1 REMOVAL FROM CHROMATIN
ASSEMBLY OF THE PRE REPLICATIVE COMPLEX
APC C CDH1 MEDIATED DEGRADATION OF CDC20 AND OTHER APC C CDH1 TARGETED PROTEI
CDT1 ASSOCIATION WITH THE CDC6 ORC ORIGIN COMPLEX
APC C CDC20 MEDIATED DEGRADATION OF MITOTIC PROTEINS
CYCLIN E ASSOCIATED EVENTS DURING G1 S TRANSITION
OLFACTORY SIGNALING PATHWAY
ANTIVIRAL MECHANISM BY IFN STIMULATED GENES
ACTIVATION OF NF KAPPAB IN B CELLS
REGULATION OF ORNITHINE DECARBOXYLASE ODC
SCFSKP2 MEDIATED DEGRADATION OF P27 P21
P53 INDEPENDENT G1 S DNA DAMAGE CHECKPOINT
P53 DEPENDENT G1 DNA DAMAGE RESPONSE
CDK MEDIATED PHOSPHORYLATION AND REMOVAL OF CDC6
SCF BETA TRCP MEDIATED DEGRADATION OF EMI1
FACTORS INVOLVED IN MEGAKARYOCYTE DEVELOPMENT AND PLATELET PRODUCTION
REGULATION OF MRNA STABILITY BY PROTEINS THAT BIND AU RICH ELEMENTS
ER PHAGOSOME PATHWAY
AUTODEGRADATION OF CDH1 BY CDH1 APC C
DESTABILIZATION OF MRNA BY AUF1 HNRNP D0
AUTODEGRADATION OF THE E3 UBIQUITIN LIGASE COP1
Stats_entropy
Wavelet_LLL_glcm_sumEntro
Wavelet_LLL_stats_entropy
GLCM_sumEntro
GLCM_entrop2
Wavelet_LLL_glcm_entrop2
Wavelet_HLL_glcm_entrop2
Stats_md
Wavelet_LLL_stats_md
Wavelet_HLL_glcm_sumEntro
Stats_std
Stats_var
Wavelet_LLL_stats_std
Wavelet_LLL_stats_var
Wavelet_LHL_glcm_clusProm
Wavelet_HHH_glcm_contrast
Wavelet_HHH_glcm_clusTend
Wavelet_HHH_glcm_clusProm
Wavelet_HLH_glcm_clusProm
Wavelet_HHH_glcm_sumEntro
Wavelet_HHH_stats_entropy
Wavelet_HHH_glcm_diffEntro
Wavelet_HHH_glcm_dissimilar
Wavelet_HHH_glcm_entrop2
Wavelet_LHL_glcm_clusTend
Wavelet_HLH_glcm_contrast
Wavelet_HLH_glcm_diffEntro
Wavelet_HLH_glcm_dissimilar
Wavelet_LLH_glcm_clusTend
Wavelet_LLL_glcm_clusTend
GLCM_clusTend
Wavelet_LLH_glcm_clusProm
Stats
Texture
Shape
Wavelet
LoG

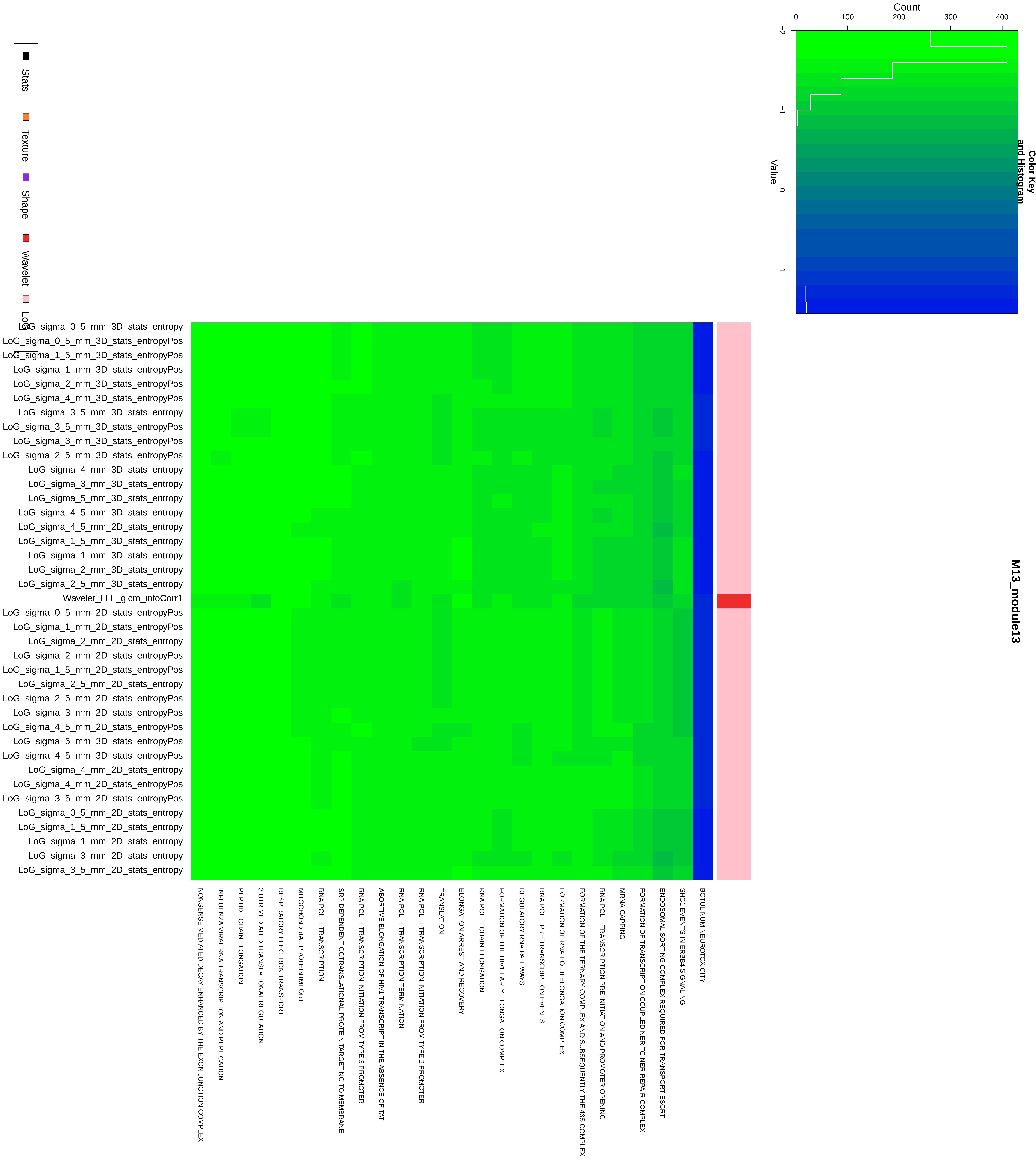

Count
Color Key and Histogram
Value
Stats
Texture
Shape
Wavelet
LoG
M13_module13
LoG_sigma_0_5_mm_3D_stats_entropy
LoG_sigma_0_5_mm_3D_stats_entropyPos
LoG_sigma_1_5_mm_3D_stats_entropyPos
LoG_sigma_1_mm_3D_stats_entropyPos
LoG_sigma_2_mm_3D_stats_entropyPos
LoG_sigma_4_mm_3D_stats_entropyPos
LoG_sigma_3_5_mm_3D_stats_entropy
LoG_sigma_3_5_mm_3D_stats_entropyPos
LoG_sigma_3_mm_3D_stats_entropyPos
LoG_sigma_2_5_mm_3D_stats_entropyPos
LoG_sigma_4_mm_3D_stats_entropy
LoG_sigma_3_mm_3D_stats_entropy
LoG_sigma_5_mm_3D_stats_entropy
LoG_sigma_4_5_mm_3D_stats_entropy
LoG_sigma_4_5_mm_2D_stats_entropy
LoG_sigma_1_5_mm_3D_stats_entropy
LoG_sigma_1_mm_3D_stats_entropy
LoG_sigma_2_mm_3D_stats_entropy
LoG_sigma_2_5_mm_3D_stats_entropy
Wavelet_LLL_glcm_infoCorr1
LoG_sigma_0_5_mm_2D_stats_entropyPos
LoG_sigma_1_mm_2D_stats_entropyPos
LoG_sigma_2_mm_2D_stats_entropy
LoG_sigma_2_mm_2D_stats_entropyPos
LoG_sigma_1_5_mm_2D_stats_entropyPos
LoG_sigma_2_5_mm_2D_stats_entropy
LoG_sigma_2_5_mm_2D_stats_entropyPos
LoG_sigma_3_mm_2D_stats_entropyPos
LoG_sigma_4_5_mm_2D_stats_entropyPos
LoG_sigma_5_mm_3D_stats_entropyPos
LoG_sigma_4_5_mm_3D_stats_entropyPos
LoG_sigma_4_mm_2D_stats_entropy
LoG_sigma_4_mm_2D_stats_entropyPos
LoG_sigma_3_5_mm_2D_stats_entropyPos
LoG_sigma_0_5_mm_2D_stats_entropy
LoG_sigma_1_5_mm_2D_stats_entropy
LoG_sigma_1_mm_2D_stats_entropy
LoG_sigma_3_mm_2D_stats_entropy
LoG_sigma_3_5_mm_2D_stats_entropy
NONSENSE MEDIATED DECAY ENHANCED BY THE EXON JUNCTION COMPLEX
INFLUENZA VIRAL RNA TRANSCRIPTION AND REPLICATION
PEPTIDE CHAIN ELONGATION
3 UTR MEDIATED TRANSLATIONAL REGULATION
RESPIRATORY ELECTRON TRANSPORT
MITOCHONDRIAL PROTEIN IMPORT
RNA POL III TRANSCRIPTION
SRP DEPENDENT COTRANSLATIONAL PROTEIN TARGETING TO MEMBRANE
RNA POL III TRANSCRIPTION INITIATION FROM TYPE 3 PROMOTER
ABORTIVE ELONGATION OF HIV1 TRANSCRIPT IN THE ABSENCE OF TAT
RNA POL III TRANSCRIPTION TERMINATION
RNA POL III TRANSCRIPTION INITIATION FROM TYPE 2 PROMOTER
TRANSLATION
ELONGATION ARREST AND RECOVERY
RNA POL III CHAIN ELONGATION
FORMATION OF THE HIV1 EARLY ELONGATION COMPLEX
REGULATORY RNA PATHWAYS
RNA POL II PRE TRANSCRIPTION EVENTS
FORMATION OF RNA POL II ELONGATION COMPLEX
FORMATION OF THE TERNARY COMPLEX AND SUBSEQUENTLY THE 43S COMPLEX
RNA POL II TRANSCRIPTION PRE INITIATION AND PROMOTER OPENING
MRNA CAPPING
FORMATION OF TRANSCRIPTION COUPLED NER TC NER REPAIR COMPLEX
ENDOSOMAL SORTING COMPLEX REQUIRED FOR TRANSPORT ESCRT
SHC1 EVENTS IN ERBB4 SIGNALING
BOTULINUM NEUROTOXICITY
