# Supplementary material for: Defining the biological basis of radiomic phenotypes in lung cancer
Source: eLife. 2017 Jul 21;6:e23421. doi: 10.7554/eLife.23421 (PMC5590809; doi:10.7554/eLife.23421)
Supplement: Table 3—source data 1. — DOI: http://dx.doi.org/10.7554/eLife.23421.014 [file elife-23421-table3-data1.docx]

| **Module** | **Strongest radiomic based pathway prediction** | **AUC** | **OS** | **ST** | **HI** |
| --- | --- | --- | --- | --- | --- |
| M1 | Wavelet_HHH_glcm_correl1 🡪  CHOLESTEROL_BIOSYNTHESIS | 0.64, *p=*0.014 |  |  |  |
| M2 | LoG_sigma_3_5_mm_2D_stats_std 🡪  AUTODEGRADATION_OF_THE_E3_UBIQUITIN_LIGASE_COP1 | 0.69, *p=*8e-4 | x | x |  |
| M3 | Wavelet_HHL_stats_min 🡪  TRAFFICKING_OF_GLUR2_CONTAINING_AMPA_RECEPTORS | 0.67, *p*=0.003 |  |  |  |
| M4 | Wavelet_HLL_stats_min 🡪  GLUTATHIONE_CONJUGATION | 0.68, *p*=9e-4 |  | x |  |
| M5 | GLCM_infoCorr2 🡪  TRAFFICKING_OF_GLUR2_CONTAINING_AMPA_RECEPTORS | 0.69, *p*=7e-4 |  | x | x |
| M6 | Wavelet_LLL_glcm_clusProm 🡪  NOTCH1_INTRACELLULAR_DOMAIN_REGULATES_TRANSCRIPTION | 0.66, *p*=0.007 |  | x | x |
| M7 | LoG_sigma_4_mm_3D_stats_entropy 🡪  RNA_POL_III_TRANSCRIPTION | 0.62, *p*=0.031 |  | x |  |
| M8 | LoG_sigma_4_mm_2D_stats_std 🡪  PYRUVATE_METABOLISM_AND_CITRIC_ACID_TCA_CYCLE | 0.72, *p*=6e-5 |  | x |  |
| M9 | Wavelet_LLL_stats_var 🡪  TRAFFICKING_OF_GLUR2_CONTAINING_AMPA_RECEPTORS | 0.64, *p*=0.020 | x |  |  |
| M10 | Shape_compactness2/Shape_sphericity 🡪  TRAF6_MEDIATED_NFKB_ACTIVATION | 0.66, *p*=0.003 |  | x | x |
| M11 | Wavelet_HLL_glcm_clusTend 🡪  PLATELET_AGGREGATION_PLUG_FORMATION | 0.69, *p*=6e-4 |  | x | x |
| M12 | Wavelet_LLL_glcm_entrop2 🡪  G0_AND_EARLY_G1 | 0.65, *p*=0.007 | x | x | x |
| M13 | LoG_sigma_4_mm_2D_stats_entropy 🡪  RNA_POL_II_TRANSCRIPTION_PRE_INITIATION_AND_PROMOTER_OPENING | 0.68, *p*=0.001 |  | x |  |

**Table S5**: Radiomic based pathway predictors with radiomic feature parameter details: GLCM=Gray-level co-occurrence matrix, sigma_*x*_mm=standard deviation of *x* mm of a Gaussian, HHH=high-high-high-pass filter, LLL=low-low-low-pass filter, HHL=high-high-low pass filter, etc. Additional information can be found in supplementary File S2.
