# Supplementary material for: Defining the biological basis of radiomic phenotypes in lung cancer
Source: eLife. 2017 Jul 21;6:e23421. doi: 10.7554/eLife.23421 (PMC5590809; doi:10.7554/eLife.23421)
Supplement: Supplementary file 1. — DOI: http://dx.doi.org/10.7554/eLife.23421.020 [file elife-23421-supp1.pdf]

We included 636 radiomic features in our analysis, which can be divided into the following groups:

1. Group 1: First-order statistics (e.g., mean, standard deviation, kurtosis). These features are based on the histogram of the voxel intensity values of the image.
2. Group 2: Shape and size based features (e.g., sphericity, maximal diameter, volume). These features are based on the 3D representation of a tumor.
3. Group 3: Textural features (e.g., entropy, gray-level non-uniformity). These features measure textural structures.
4. Group 4: Wavelet features (e.g., wavelet energy, wavelet median). To calculate these features, a wavelet decomposition is first applied to the image before computing Group 1-3 features.
5. Group 5: Laplace of Gaussian (LoG) features (e.g., LoG skewness, LoG uniformity). To calculate these features, an LoG filter is applied to the image first, which results in highlighted edges. Next we compute Group 1 features.

Group 1-4 features were calculated as specified by Aerts et al. *(1)*, however we added new textural features to Group 3, which are based on a gray-level size-zone matrix (GLSZM) *(2)* as defined below, and we added the new Group 5 of LoG features. While those new textural feature quantify structures of same gray-level in the image, LoG features should highlight edges of the tumor. Our final dataset consisted of 15 Group 1 features, 13 Group 2 features, 44 Group 3 features, 384 Group 4 features, and 180 LoG features summing up to 636 radiomic features. As described in more detail by Aerts et al. *(1)*, features were calculated in 3D in every possible cube slice and averaged over all slices.

Group 3, Gray-level size-zone matrix (GLSZM)

A flat zone is defined as a group of connecting pixels of same gray-level. Gray-level size-zone matrices store information about the sizes of different flat zones found in the image. A GLZSM is defined as a matrix $p(i,j)$, where an element (i,j) is the frequency of a flat zone size j of gray-level i. The following example demonstrates how to construct $p(i,j)$ by a 5x5 image matrix I containing (in this example five) discrete gray-levels.

$$I = \begin{matrix} 5 & 2 & 5 & 4 & 4 \\ 3 & 3 & 3 & 1 & 3 \\ 2 & 1 & 1 & 1 & 3 \\ 4 & 2 & 2 & 2 & 3 \\ 3 & 5 & 3 & 3 & 2 \end{matrix} \qquad p = \begin{matrix} 0 & 0 & 0 & 1 & 0 \\ 2 & 0 & 1 & 0 & 0 \\ 1 & 1 & 2 & 0 & 0 \\ 1 & 1 & 0 & 0 & 0 \\ 3 & 0 & 0 & 0 & 0 \end{matrix}$$

Our GLSZM features are defined by use of $p(i, j)$. In the following, the feature definitions are listed, given that N_g is the number of discrete gray-levels, N_r is the number of different flat zones, and N_p is the number of voxels in the image.

- **Small Area Emphasis (SAE)**
  - $SAE = \frac{\sum_{i=1}^{N_g}\sum_{j=1}^{N_r}\left[\frac{p(i,j)}{j^2}\right]}{\sum_{i=1}^{N_g}\sum_{j=1}^{N_r}p(i,j)}$
- **Large Area Emphasis (LRE)**
  - $LAE = \frac{\sum_{i=1}^{N_g}\sum_{j=1}^{N_r}j^2 p(i,j)}{\sum_{i=1}^{N_g}\sum_{j=1}^{N_r}p(i,j)}$
- **Intensity Variability (IV)**
  - $IV = \frac{\sum_{i=1}^{N_g}\left[\sum_{j=1}^{N_r}p(i,j)\right]^2}{\sum_{i=1}^{N_g}\sum_{j=1}^{N_r}p(i,j)}$
- **Size Zone Variability (SZV)**
  - $SZV = \frac{\sum_{j=1}^{N_r}\left[\sum_{i=1}^{N_g}p(i,j)\right]^2}{\sum_{i=1}^{N_g}\sum_{j=1}^{N_r}p(i,j)}$
- **Zone Percentage (ZP)**
  - $ZP = \sum_{i=1}^{N_g}\sum_{j=1}^{N_r}\frac{p(i,j)}{N_p}$
- **Low Intensity Emphasis (LIE)**
  - $LIE = \frac{\sum_{i=1}^{N_g}\sum_{j=1}^{N_r}\left[\frac{p(i,j)}{i^2}\right]}{\sum_{i=1}^{N_g}\sum_{j=1}^{N_r}p(i,j)}$
- **High Intensity Emphasis (HIE)**
  - $HIE = \frac{\sum_{i=1}^{N_g}\sum_{j=1}^{N_r}i^2 p(i,j)}{\sum_{i=1}^{N_g}\sum_{j=1}^{N_r}p(i,j)}$

- **Low Intensity Small Area Emphasis (LISAE)**
  - $LISAE = \frac{\sum_{i=1}^{N_g}\sum_{j=1}^{N_r}\left[\frac{p(i,j)}{i^2 j^2}\right]}{\sum_{i=1}^{N_g}\sum_{j=1}^{N_r} p(i,j)}$
- **High Intensity Small Area Emphasis (HISAE)**
  - $HISAE = \frac{\sum_{i=1}^{N_g}\sum_{j=1}^{N_r}\left[\frac{p(i,j)i^2}{j^2}\right]}{\sum_{i=1}^{N_g}\sum_{j=1}^{N_r} p(i,j)}$
- **Low Intensity Large Area Emphasis (LILAE)**
  - $LILAE = \frac{\sum_{i=1}^{N_g}\sum_{j=1}^{N_r}\left[\frac{p(i,j)j^2}{i^2}\right]}{\sum_{i=1}^{N_g}\sum_{j=1}^{N_r} p(i,j)}$
- **High Intensity Large Area Emphasis (HILAE)**
  - $HILAE = \frac{\sum_{i=1}^{N_g}\sum_{j=1}^{N_r} p(i,j)i^2 j^2}{\sum_{i=1}^{N_g}\sum_{j=1}^{N_r} p(i,j)}$

Group 5, Laplace of Gaussian (LoG)

Applying a Laplace to an image highlights areas of rapid intensity changes, and is hence used to detect edge structures. Laplace of Gaussian filters first apply a Gaussian before computing the Laplace to smooth the image. Let (x, y) be the pixel intensity of the image at position x and y, then the 2D kernel LoG with mean zero and standard deviation σ is defined as

$$LoG(x, y) = -\frac{1}{\pi\sigma^4}\left[1 - \frac{x^2+y^2}{2\sigma^2}\right]e^{-\frac{x^2+y^2}{2\sigma^2}}.$$

After the LoG filtering, we calculated Group 1 features. We varied σ from 0.5mm to 5mm by an increment of 0.5mm for every Group 1 features to highlight fine and coarse textures. To calculate LoG mean, LoG uniformity, and LoG entropy, negative pixel values were set to zero. We applied above 2D LoG method to every dimension in 3D.

References

1. H. J. W. L. Aerts, E. R. Velazquez, R. T. H. Leijenaar, C. Parmar, P. Grossmann, S. Cavalho, J. Bussink, R. Monshouwer, B. Haibe-Kains, D. Rietveld, F. Hoebers, M. M. Rietbergen, C. R. Leemans, A. Dekker, J. Quackenbush, R. J. Gillies, P. Lambin, Decoding tumour phenotype by noninvasive imaging using a quantitative radiomics approach, *Nat. Commun.* **5** (2014), doi:10.1038/ncomms5006.

2. G. Thibault, J. Angulo, F. Meyer, Advanced Statistical Matrices for Texture Characterization: Application to Cell Classification, *IEEE Trans. Biomed. Eng.* **61**, 630–637 (2014).
