# Supplementary material for: Defining the biological basis of radiomic phenotypes in lung cancer
Source: eLife. 2017 Jul 21;6:e23421. doi: 10.7554/eLife.23421 (PMC5590809; doi:10.7554/eLife.23421)
Supplement: Supplementary file 2. — DOI: http://dx.doi.org/10.7554/eLife.23421.022 [file elife-23421-supp2.pdf]

Additional information

Identifying Molecular Phenotypes in Lung Cancer by Integrating Radiomics and Genomics

Patrick Grossmann[*12i], Olya Grove[†3i], Nehme El-Hachem[4], Emmanuel Rios Velazquez[1], Ralph T.H. Leijenaar[6], Chintan Parmar[16], Benjamin Haibe-Kains[5], Philippe Lambin[6], Robert J. Gillies [‡3i], and Hugo J.W.L. Aerts[§127i]

[4] *Institut de recherches cliniques de Montreal, Montreal, Quebec, Canada.*
[1] *Departments of Radiation Oncology, Dana-Farber Cancer Institute, Harvard Medical School, Boston, MA, 02215-5450, USA.*
[2] *Department of Biostatistics & Computational Biology, Dana-Farber Cancer Institute, Harvard School of Public Health, Boston, MA, 02215-5450, USA.*
[5] *Princess Margaret Cancer Centre, University Health Network, Toronto, Ontario, Canada.*
[6] *Department of Radiation Oncology (MAASTRO), Research Institute GROW, Maastricht University, 6229ET Maastricht, Netherlands.*
[7] *Department of Radiology Brigham and Women's Hospital, Harvard Medical School, Boston, 02215-5450, MA, USA.*
[3] *Department of Cancer Imaging and Metabolism, H. Lee Moffitt Cancer Center and Research Institute, Tampa, FL 33612, USA.*
[i] *Equally contributed*

June 22, 2017

[*] patrick@jimmy.harvard.edu
[†] olya.grove@moffitt.org
[‡] robert.gillies@moffitt.org
[§] hugo@jimmy.harvard.edu

Contents

1 File S4: Additional information 3
1.1 Modules showing the relationships of radiomics and biology 4
1.2 Clinical value of modules 4
1.3 Statistical libraries used for the analysis 4

1 File S4: Additional information

1.1 Modules showing the relationships of radiomics and biology

Our analysis revealed 13 modules of coherently expressed radiomic features and molecular pathways. Supplementary file SII contains heatmaps of hierarchical Ward linkage clusterings on the corresponding normalized enrichemnt scores (NESs) for each of the modules including the complete sets of radiomic feature names and pathway names. All NESs have been transformed to Z-scores before clustering. Here we provide additional information about the module analysis.

1.2 Clinical value of modules

Modules have been associatd with clinical information (prognosis, histology, and stage) by combining p-values of random permutation tests on Lung1 and Lung2 in a meta-analysis Z-transformation [1]. The underlying p-values are given in the following tables.

1.3 Statistical libraries used for the analysis

All statistical analyses were carried out using the R software (R Core Team, Vienna, Austria) version 3.1.0 on a Linux environment [2]. The following R and Bioconductor [3] package versions were used:

	1	2	3	4	5	6	7	8	9	10	11	12	13
Z	0.40	0.01	0.43	0.07	0.11	0.23	0.18	0.45	0.01	0.66	0.11	0.00	0.34
Lung1	0.54	0.02	0.43	0.14	0.12	0.30	0.25	0.56	0.02	0.79	0.19	0.01	0.44
Lung2	0.14	0.09	0.50	0.07	0.34	0.24	0.17	0.20	0.13	0.13	0.12	0.04	0.18

Table 1: P-values of random permutation tests of mean CIs of survival times in each module.

	1	2	3	4	5	6	7	8	9	10	11	12	13
Z	0.24	0.12	0.58	0.07	0.01	0.02	0.16	0.64	0.81	0.01	0.02	0.05	0.13
Lung1	0.23	0.21	0.44	0.20	0.01	0.05	0.29	0.70	0.91	0.04	0.03	0.09	0.25
Lung2	0.47	0.09	0.86	0.01	0.13	0.04	0.08	0.33	0.11	0.01	0.19	0.11	0.07

Table 2: P-values of random permutation tests of mean Kruskal-Wallis chi square statistics in each module with respect to histology.

	1	2	3	4	5	6	7	8	9	10	11	12	13
Z	0.62	0.00	0.25	0.01	0.00	0.00	0.00	0.01	0.19	0.01	0.00	0.00	0.00
Lung1	0.73	0.00	0.23	0.01	0.00	0.00	0.00	0.01	0.34	0.04	0.00	0.00	0.00
Lung2	0.21	0.04	0.50	0.22	0.05	0.02	0.10	0.17	0.07	0.02	0.29	0.09	0.02

Table 3: P-values of random permutation tests of mean Kruskal-Wallis chi square statistics in each module with respect to stage.

- R version 3.1.0 (2014-04-10), `x86_64-unknown-linux-gnu`
- Base packages: base, datasets, graphics, grDevices, grid, methods, parallel, splines, stats, stats4, utils
- Other packages: affy 1.44.0, annotate 1.44.0, AnnotationDbi 1.28.1, Biobase 2.26.0, BiocGenerics 0.12.1, biomaRt 2.22.0, bitops 1.0-6, DBI 0.3.1, eisa 1.18.0, FactoMineR 1.28, genefu 1.15.1, GenomeInfoDb 1.2.3, GGally 0.5.0, ggplot2 1.0.0, GO.db 3.0.0, gplots 2.15.0, graph 1.44.1, GSEABase 1.28.0, gsubfn 0.6-6, GSVA 1.14.1, igraph 0.7.1, inSilicoDb 2.2.0, IRanges 2.0.1, isa2 0.3.3, jetset 1.6.0, lsa 0.73, mclust 4.4, MetaGx 0.0.2, mRMRe 2.0.5, org.Hs.eg.db 3.0.0, plyr 1.8.1, prodlim 1.5.1, proto 0.3-10, RadioGx 1.7, RamiGO 1.12.0, RCurl 1.95-4.5, RCytoscape 1.16.0, reshape2 1.4.1, rjson 0.2.15, R.methodsS3 1.6.1, R.oo 1.18.0, RSQLite 1.0.0, R.utils 1.34.0, S4Vectors 0.4.0, SnowballC 0.5.1, survcomp 1.16.0, survival 2.37-7, VennDiagram 1.6.9, WriteXLS 3.5.1, XML 3.98-1.1, XMLRPC 0.3-0, xtable 1.7-4
- Loaded via a namespace (and not attached): affyio 1.34.0, amap 0.8-12, BiocInstaller 1.16.1, bootstrap 2014.4, car 2.0-22, Category 2.32.0, caTools 1.17.1, cluster 1.15.3, colorspace 1.2-4, digest 0.6.6, gdata 2.13.3, genefilter 1.48.1, gtable 0.1.2, gtools 3.4.1, KernSmooth 2.23-13, labeling 0.3, lattice 0.20-29, lava 1.3, leaps 2.9, MASS 7.3-35, Matrix 1.1-4, munsell 0.4.2, nnet 7.3-8, png 0.1-7, preprocessCore 1.28.0, RBGL 1.42.0, RColorBrewer 1.1-2, Rcpp 0.11.3, reshape 0.8.5, rmeta 2.16, scales 0.2.4, scatterplot3d 0.3-35, stringr 0.6.2, SuppDists 1.1-9.1, survivalROC 1.0.3, tcltk 3.1.0, tools 3.1.0, zlibbioc 1.12.0

References

[1] M. C. Whitlock. Combining probability from independent tests: the weighted Z-method is superior to Fisher's approach. *Journal of evolutionary biology*, 18(5):1368–1373, September 2005.

[2] R Core Team. *R: A Language and Environment for Statistical Computing.* R Foundation for Statistical Computing, Vienna, Austria, 2013. ISBN 3-900051-07-0.

[3] Robert Gentleman, Vincent Carey, Douglas Bates, Ben Bolstad, Marcel Dettling, Sandrine Dudoit, Byron Ellis, Laurent Gautier, Yongchao Ge, Jeff Gentry, Kurt Hornik, Torsten Hothorn, Wolfgang Huber, Stefano Iacus, Rafael Irizarry, Friedrich Leisch, Cheng Li, Martin Maechler, Anthony Rossini, Gunther Sawitzki, Colin Smith, Gordon Smyth, Luke Tierney, Jean Yang, and Jianhua Zhang. Bioconductor: open software development for computational biology and bioinformatics. *Genome Biology*, 5(10):R80–16, 2004.
