# Supplementary material for: Defining the biological basis of radiomic phenotypes in lung cancer
Source: eLife. 2017 Jul 21;6:e23421. doi: 10.7554/eLife.23421 (PMC5590809; doi:10.7554/eLife.23421)
Supplement: Supplementary file 3. — DOI: http://dx.doi.org/10.7554/eLife.23421.023 [file elife-23421-supp3.pdf]

Automated pathology assessment

Positive Pixel Count. PPC was obtained by running our customized algorithm (PPC v9) in Aperio eSlide Manager, the database used for digital pathology "eSlides" storage and management. The eSlide manager also handles the positive pixel count analysis jobs and stores the data associated with each slide. This data can then be exported and sorted in Microsoft Excel. The PPC algorithm determines positive pixels of an IHC slide against a Region of Interest (ROI) annotated within ImageScope. ImageScope is an eSlide manager partner application that opens the ".svs" file format of the eSlides. From ImageScope, annotations for ROIs can be made and observed as well as the masks for positive/negative pixel counts of the tissue. Snapshots at the various 0.6X, 2.0X, and 5.0X were taken within ImageScope and saved in TIF image format.

Cluster Counts Analysis. The cluster count analysis was developed in separate software Definiens Developer 2.5. The algorithm was developed from scratch within the software and run across each tissue image with a few using manual ROIs that matched any ROIs used in the PPC analysis. Data was the exported and sorted within Microsoft Excel. Algorithm was developed by Joseph Johnson and Jonathan Nguyen.

Digital Pathology Slide Scanning. The histology slides were dropped off by author OG and scanned into the eSlide Manager database using our Aperio Scanscope XT slide scanner at 20X magnification.
